# Structure–Activity Relationship of Piplartine and Synthetic Analogues against *Schistosoma mansoni* and Cytotoxicity to Mammalian Cells

**DOI:** 10.3390/ijms19061802

**Published:** 2018-06-19

**Authors:** Yuri Campelo, Alicia Ombredane, Andreanne G. Vasconcelos, Lucas Albuquerque, Daniel C. Moreira, Alexandra Plácido, Jefferson Rocha, Harold Hilarion Fokoue, Lydia Yamaguchi, Ana Mafud, Yvonne P. Mascarenhas, Cristina Delerue-Matos, Tatiana Borges, Graziella A. Joanitti, D. R. Daniel Arcanjo, Massuo J. Kato, Selma A. S. Kuckelhaus, Marcos P. N. Silva, Josué de Moraes, José Roberto S. A. Leite

**Affiliations:** 1 Núcleo de Pesquisa em Biodiversidade e Biotecnologia, Biotec, Universidade Federal do Piauí, UFPI, Parnaíba-PI, 64202-020 Brazil; yd.dias@hotmail.com; 2 Programa de Pós-Graduação em Biotecnologia, RENORBIO, Ponto focal Universidade Federal do Piauí, UFPI, Teresina, PI, 64049-550, Brazil; jeffersonbiotec@gmail.com; 3 Instituto de Educação Superior do Vale do Parnaíba, FAHESP/IESVAP, Parnaíba-PI, 64212-790, Brazil; 4 Laboratório de Nanobiotecnologia, Instituto de Biologia, Campus Darcy Ribeiro, Universidade de Brasília, UnB, Brasília-DF 70910-900, Brazil; aliciaombredane@gmail.com (A.O.); bygra1@gmail.com (G.A.J.); 5 Área de Morfologia, Faculdade de Medicina, Universidade de Brasília, UnB, Brasília-DF 70910-900, Brazil; andreannegv@gmail.com (A.G.V.); danielcarmor@gmail.com (D.C.M.); selmask@gmail.com (S.A.S.K.); 6 Laboratorio de Imunologia, Faculdade de Medicina, Universidade de Brasília, UnB, Brasília-DF 70910-900, Brazil; lucasfriaca@hotmail.com (L.A.); tatianakarlab@gmail.com (T.B.); 7 LAQV/REQUIMTE, GRAQ, Instituto Superior de Engenha do Porto, ISEP, Porto 4200-072, Portugal; alexandra.placido@gmail.com (A.P.); cmm@isep.ipp.pt (C.D.-M.); 8 Laboratório de Avaliação e Síntese de Substâncias Bioativas, Universidade Federal do Rio de Janeiro, CCS, Cidade Universitária, Rio de Janeiro-RJ 21941-902, Brasil; hfokoue@gmail.com; 9 Instituto de Química, Universidade de São Paulo, São Paulo-SP 01005-010, Brazil; lydyama@gmail.com (L.Y.); majokato@iq.usp.br (M.J.K.); 10 Instituto de Física de São Carlos, Universidade de São Paulo-SP 01005-010, Brazil; carolmafud@gmail.com (A.M.); yvonne@ifsc.usp.br (Y.P.M.); 11 Núcleo de Pesquisas em Plantas Medicinais, NPPM, Universidade Federal do Piauí, UFPI, Parnaíba-PI 64202-020, Brazil; daniel.arcanjo@ufpi.edu.br; 12 Núcleo de Pesquisa em Doenças Negligenciadas, Universidade de Guarulhos, Guarulhos-SP 07023-070, Brazil; marcos.p.bio@gmail.com (M.P.N.S.); josuem@usp.br (J.d.M.)

**Keywords:** piplartine, analogues, *Schistosoma mansoni*, cytotoxicity

## Abstract

Schistosomiasis, caused by helminth flatworms of the genus *Schistosoma*, is an infectious disease mainly associated with poverty that affects millions of people worldwide. Since treatment for this disease relies only on the use of praziquantel, there is an urgent need to identify new antischistosomal drugs. Piplartine is an amide alkaloid found in several *Piper* species (Piperaceae) that exhibits antischistosomal properties. The aim of this study was to evaluate the structure–function relationship between piplartine and its five synthetic analogues (19A, 1G, 1M, 14B and 6B) against *Schistosoma mansoni* adult worms, as well as its cytotoxicity to mammalian cells using murine fibroblast (NIH-3T3) and BALB/cN macrophage (J774A.1) cell lines. In addition, density functional theory calculations and in silico analysis were used to predict physicochemical and toxicity parameters. Bioassays revealed that piplartine is active against *S. mansoni* at low concentrations (5–10 µM), but its analogues did not. In contrast, based on 3-(4,5-dimethylthiazol-2-yl)-2,5-diphenyltetrazolium bromide (MTT) and flow cytometry assays, piplartine exhibited toxicity in mammalian cells at 785 µM, while its analogues 19A and 6B did not reduce cell viability at the same concentrations. This study demonstrated that piplartine analogues showed less activity against *S. mansoni* but presented lower toxicity than piplartine.

## 1. Introduction

Schistosomiasis, caused by helminth flatworms of the genus *Schistosoma*, is a chronic and often painful disease mainly associated with poverty that affects more than 200 million people worldwide, resulting in major economic and personal impacts from the years of healthy life lost to morbidity [1]. Recent global burden of disease estimates indicate that approximately 10,000 schistosomiasis-related deaths occur each year [2]. Moreover, the current estimate of yearly disability adjusted life-years (DALYs) for schistosomiasis is 3.4 million [3]. Three species (*Schistosoma mansoni*, *Schistosoma haematobium* and *Schistosoma japonicum*) account for the majority of human infections. The major aetiological agent of human schistosomiasis is *S. mansoni* and intestinal schistosomiasis caused by this species is present in Africa, the Middle East, the Caribbean, and South America [4].

Human treatment with praziquantel is playing a central role in the control and prevention of schistosomiasis, being the only effective drug currently available [5]. However, praziquantel does not prevent re-infection and its continued extensive use may result in the future emergence of drug-resistant parasites. Having a single drug to treat a disease that affects millions of people is a real concern and, consequently, it is imperative to develop new effective antischistosomal drugs [6].

The use of natural products has a long tradition in medicine, and they have proven to be an important source of lead compounds in the development of new drugs. In order to provide new compounds for use in drug development to control schistosomiasis, the search for anthelmintic compounds, mainly from natural sources, has intensified in recent years [7,8,9,10,11]. Indeed, as reviewed elsewhere, natural compounds have been recognized as promising candidates for antischistosomal drugs [12].

In the north-eastern region of Brazil, several plants with active metabolites have been discovered, among them is the *Piperaceae* family, which includes several compounds with antimicrobial activities [13]. Piplartine (3,4,5-trimethoxycinnamoyl-*N*-dihydropyridin-2-one), also known as piperlongumine were described in several species of *Piper* genus, and anti-inflammatory, antitumor, antifungal, and antiparasitic effects were reported (for review see [14]). Our group previously demonstrated that piplartine at 10 μM possesses in vitro schistosomicidal activity against *S. mansoni* schistosomula and adult worms [15,16]. We also showed that piplartine caused morphological alterations in the tegument of parasites in a concentration-dependent manner. The antischistosomal effect of piplartine in association with other molecules (e.g., praziquantel) had also been described [17,18].

The activity of a molecule is characterised not only by its biological activity against microorganisms, but also by its toxicity in various mammalian cells. Toxicity studies are, therefore, performed prior to the administration of a molecule to humans. In order to establish a relationship between the chemical structure of piplartine and its biological effects, in this study new analogues of piplartine were synthesised and evaluated against adult *S. mansoni* ex vivo. The cytotoxicity of compounds was also evaluated on murine fibroblast (NIH-3T3) and BALB/cN macrophage (J774A.1) cell lines. Additionally, we also performed theoretical calculations and ultraviolet–visible absorption spectra to characterize the obtained compounds. Furthermore, physicochemical and toxicological parameters of piplartine and its derivatives were obtained by a computer-aided prediction.

## 2. Results

### 2.1. Theoretical Calculations and Ultraviolet–Visible Absorption Spectra

**Figure 1:**
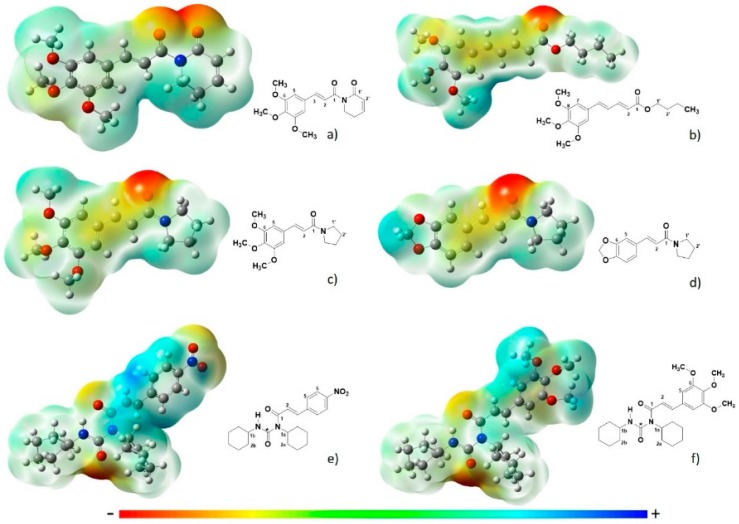
Electron density: (**a**) piplartine; (**b**) 19A; (**c**) 1G; (**d**) 14B; (**e**) 6B and (**f**) 1M. The colours represent negative (red) and positive (blue). The dipole moments of all compounds in debye values are as follows: piplartine: 5.4728337; 1G: 1.9658996; 1M: 4.4568136; 6B: 4.0062111; 14B: 3.9403826; 19A: 3.7385640.

**Figure 2:**
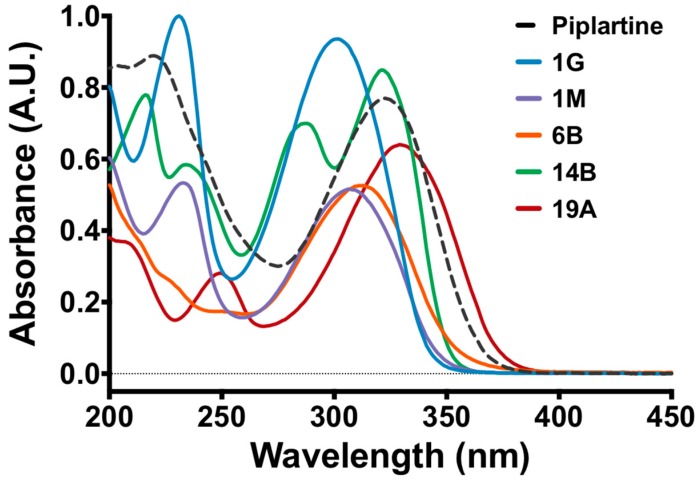
Ultraviolet and visible light absorption spectra of piplartine and its analogues in arbitrary units (A.U.).

The electron density data for the tested molecules are shown in Figure 1, and indicate that piplartine had the highest dipole moment (5.47). Regions coloured red tended to have a more negative charge, being polar, while those in blue were more positive. Figure 2 represents the ultraviolet–visible (UV–Vis) light absorption spectrum of piplartine and its analogues, showing maximum absorbance from 280 to 400 nm, with dislocation at the bands of the analogues in relation to the original molecule.

### 2.2. In Silico Studies Information

To investigate the differences between piplartine and its analogues, a structural and physicochemical analysis was performed to calculate the van der Waals surface area (vdW), molecular electrostatic potential (MEP) surface, molecular lipophilicity potential (MLP) and polar surface area (PSA), Log*_P_* values, predicted solubility, and molecular weight of the compounds, using the ChemAxon method. The results are shown in Table 1. Despite significant differences in surface area, the most significant difference between the compounds was in the topological polarised surface area (TPSA). TPSA of a molecule was defined as the surface sum over all polar atoms and is based simply on the summation of polar fragments representing tabulated surface contributions, i.e., bonding patterns of a molecule [19]. The fragments present in the molecules were compared with a library of substructure fragments to determine the toxic characteristics of each of the studied molecules as well as to predict toxicity activity. The results of this analysis are shown in Table 2, and indicate that piplartine shows greater toxicity and increased ability to bind to nuclear receptors than its analogues, effects that can be related to its cytotoxic effects observed during in vitro tests in mammalian cells. In addition, the Ames mutagenicity prediction revealed only positive for piplartine.

### 2.3. Cytotoxicity Assay

**Figure 3:**
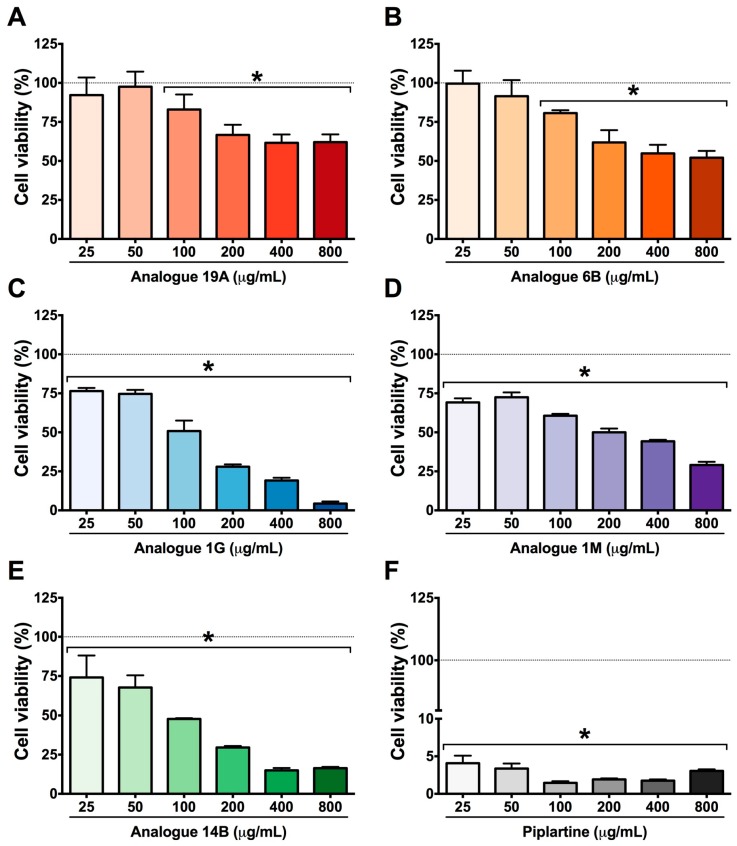
Cytotoxicity evaluation using the MTT method in murine fibroblast (NIH3T3) cells after exposure for 24 h to piplartine analogues (**A**) 19A (72–2331 μM), (**B**) 6B (62–1998 μM), (**C**) 1G (85–2738 μM), (**D**) 1M (56–1796 μM), (**E**) 14B (101–3250 μM) and (**F**) piplartine (78–2514 μM). Molecules were used at the same concentrations range at μg/mL for all samples (25–800). Dimethyl sulfoxide (DMSO) was used as a negative control. The values are expressed as mean ± SEM. * *p* < 0.05 vs. DMSO control group. The dotted lines mark the 100% viability level.

The cytotoxicity of piplartine and its analogues was evaluated by MTT assay in a murine fibroblast (NIH-3T3) cell line at concentrations from 25 to 800 µg/mL. The results demonstrated that piplartine decreased cell viability after 24 h of exposure, exhibiting considerable toxicity (Figure 3). Analogues 1G, 14B, and 1M promoted a considerable reduction in cell viability at concentrations of 400 and 800 μg/mL when compared with piplartine. Furthermore, 1G and 14B presented the highest pD_2_ and E_max_ values when compared with the other analogues (Table 3). However, analogues 19A and 6B did not exert significant effects on mammalian cell viability at the tested concentrations when compared with the effects of piplartine (Figure 3). The pD_2_ values of 19A and 6B were estimated based on their respective E_max_ values, which were lower than 50% (Table 3), indicating lower cytotoxicity than piplartine and the other analogues. Thus, among the tested compounds, piplartine presented the highest toxicity. The dimethylsulphoxide (DMSO) vehicle control displayed no effect on cell viability.

**Figure 4:**
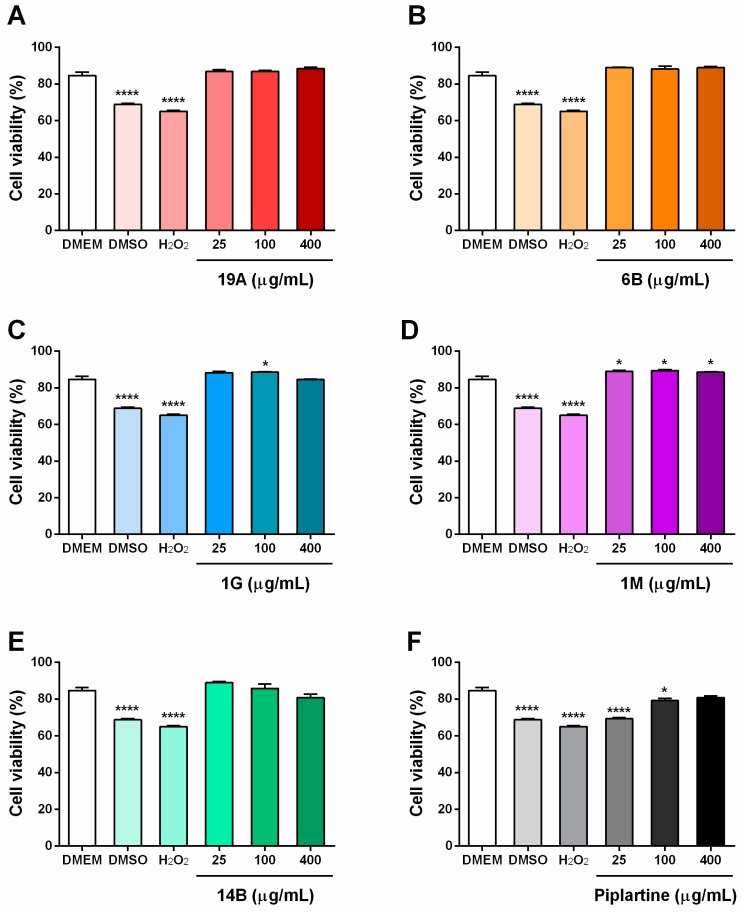
Cytotoxicity evaluation in the mouse BALB/cN macrophage (J774A.1) cell line following exposure for 24 h to piplartine analogues (**A**) 19A (72, 291 and 1165 μM), (**B**) 6B (62, 249 and 999 μM), (**C**) 1G (85, 342 and 1369 μM), (**D**) 1M (56, 224 and 898 μM), (**E**) 14B (101, 406 and 1625 μM) and (**F**) piplartine (78, 314 and 1257 μM). Molecules were used at the same concentrations range at μg/mL for all samples (25, 100 and 400). Dulbecco’s Modified Eagle Medium (DMEM) was used as a negative control. Cells were analysed by flow cytometry (20,000 events/sample). The values are expressed as mean ± SEM. * *p <* 0.05 and **** *p <* 0.0001 vs. DMEM control group.

The cytotoxicity of piplartine and its analogues was also evaluated by flow cytometry in a mouse BALB/cN macrophage (J774A.1) cell line at concentrations of 25, 100 and 400 µg/mL with exposure for 24 h (Figure 4). The DMSO vehicle control caused a significant (*p <* 0.05) reduction in cell viability, similar to the hydrogen peroxide (H_2_O_2_) cell death control. Piplartine significantly (*p <* 0.05) affected cell viability at concentrations of 25 and 100 µg/mL when compared with a DMEM untreated control. Analogue 1M, at all tested concentrations, and analogue 1G at 100 µg/mL (342 µM) significantly altered (*p <* 0.05) cell viability in comparison with the negative control, but not in a toxic manner. Other analogues did not show significant effects on cell viability. Interestingly, treatment with piplartine and its analogues appeared to reduce the cytotoxicity of the DMSO vehicle on J774A.1 cells.

**Figure 5:**
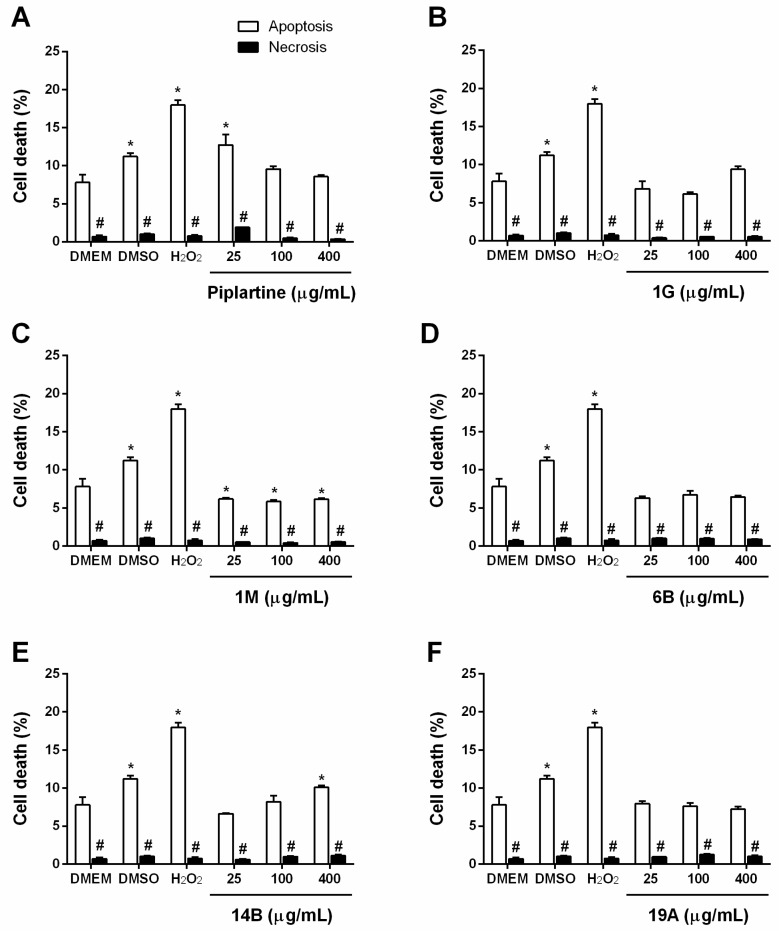
Evaluation of the cell death mechanism mediated by (**A**) piplartine (78, 314 and 1257 μM) and its analogues (**B**) 1G (85, 342 and 1369 μM), (**C**) 1M (56, 224 and 898 μM), (**D**) 6B (62, 249 and 999 μM), (**E**) 14B (101, 406 and 1625 μM) and (**F**) 19A (72, 291 and 1165 μM) in J774A.1 cells following treatment for 24 h at the same concentrations range at μg/mL for all samples (25, 100 and 400) using annexin-V FITC (apoptosis marker) and propidium iodide (PI, necrosis marker) staining. Cells were analysed by flow cytometry (20,000 events/sample). The values are expressed as mean ± SEM. * *p* < 0.05 vs. DMEM untreated control group. # *p <* 0.05 vs. apoptosis staining from each respective group.

In the same cell viability assay, the percent of non-viable cells was analysed regarding the cytotoxic mechanism in mammalian cells using annexin-V conjugated to green-fluorescent FITC and propidium iodide (PI) staining to distinguish apoptotic cells (annexin-V FITC) from necrotic cells (PI) by flow cytometry (Figure 5 and Figure S2). As expected, the control of cell death by apoptosis (H_2_O_2_) showed a significant (*p <* 0.05) increase in annexin-V FITC fluorescence intensity compared with the DMEM negative control. A significant (*p <* 0.05) increase in annexin-V FITC staining was observed in J774A.1 cells following treatment with piplartine 25 μg/mL (785 µM) and analogue 14B at 400 μg/mL (1625 µM) when compared with the DMEM negative control. Furthermore, significant (*p <* 0.05) differences between the annexin-V FITC and PI fluorescent signals were observed for each group of J774A.1 cells treated with piplartine and its analogues, indicating that apoptosis was the principal mechanism of cell death. 

### 2.4. In Vitro Experiments in Adult Worms of S. mansoni 

The activity of the amide piplartine and its analogues against adult worms of *S. mansoni* aged 49 days was most prominent at the concentration of 10 µM and incubation time of 96 h (Table 4). The amide piplartine showed activity against the parasite 24 h after incubation, while its analogues showed no activity at the tested concentrations when compared with piplartine, as noted in Table S1.

## 3. Discussion

Schistosomiasis is a global public health problem, and the search is ongoing for drugs to treat this neglected disease and meet the requirements of the standard treatment, praziquantel. Because it is a safe medication when administered to children, adults, the elderly and pregnant women, with low toxicity and few side effects, the use of praziquantel has been increasing, despite the number of patients who are resistant to the drug. Thus, the development of new anthelmintic compounds to treat schistosomiasis has become a major research objective [6,20].

Natural products represent an alternative to conventional therapeutic compounds because of the diversity in molecules and biological activities that these products present. Biodiversity results a large number of compounds with activities that are as yet unknown. Despite advancements in biotechnology, genomics and medicinal chemistry, the discovery of new drugs for the treatment of schistosomiasis remains challenging, since in addition to biological activity, drugs are required to have low toxicity [6,21]. In this context, the objective of this work was to study the schistosomicidal activity and cytotoxicity of piplartine and its synthetic analogues, and to compare the analogues to the parent compound.

An alternative strategy to minimise drug resistance is the chemical synthesis of established drugs or natural compounds with activities previously described in the literature, where chemical-structural modifications are made in functional groups that may be responsible for biological activity [6]. These modifications can increase, decrease or abolish biological activity; in some instances, these changes can promote decreased toxicity, but with an associated loss of biological activity. The structural changes made to piplartine to produce the analogues in this study resulted in a loss of activity against *S. mansoni* and a decrease in toxicity. Studies show that phenotypic analysis remains the optimal approach to the screening of anthelmintics as it is a simple and low-cost, qualitative strategy, widely used in in vitro testing [22,23].

Several studies have demonstrated the activity of a range of natural products against *S. mansoni*, although the mechanism of action of these compounds remains unknown, such as, piplartine, which is highly active against this parasite [15]. Previous results have shown that piplartine promotes changes in motility, instigating the death of the parasite, and that no difference in response was observed between male and female parasites [15]. According to the theoretical data shown in Figure 1, piplartine presents a higher electron density around the molecule, with a dipole moment of 5.47, which may influence its anthelmintic activity.

It is necessary for a drug to go to the market that the drug has good ADMET (absorption, distribution, metabolism, excretion, toxicity) properties. Drug toxicology is one of the crucial research fields in the preclinical study. Toxicity is a leading cause of attrition at all stages of drug development. In silico prediction of compound toxicity which can reduce the expenses of the company and save a lot of time has attracted considerable attention [24].

Compound 1G did not exhibit antischistosome activity at the concentrations tested. As shown in Figure 1, this compound had a low electron density, presenting a very low dipole moment when compared with piplartine. Changes in the functional groups of a molecule can influence its biological activity, and in this compound, modifications were observed in the dihydropiperidinone ring, which was replaced by piperidinyl [25].

Compounds 14B and 19A have similar electron densities, based on their dipole moment values, which are close to that of piplartine. The 19A molecule has a double bond between the rings that underwent modifications, as well as a change in the aliphatic chain between these two rings. Compound 14B underwent a dihydropiperidinone modification, being replaced with a piperidinyl ring, and in the trimethoxybenzene moiety, substitution with a benzodioxol ring quashed the schistosomicidal activity of this derivative [25]. These compounds have high electron densities and are highly similar to piplartine but are large and bulky compounds when compared with their prototype. These features may explain their lack of interaction with enzymes and ion channel receptors in the parasite, promoting the loss of biological activity against *S. mansoni* when compared with piplartine, whereas piplartine at low concentrations promotes alteration of the parasite tegument. Compound 14B has a double bond between the two altered portions, thus increasing the distance between the two rings forming the compound, which may have influenced the loss of this biological activity.

Compounds 1M and 6B present electronic densities similar to piplartine, according to Figure 1, but did not present biological activity against the *S. mansoni* parasite. This loss in activity may be related to the large size of these molecules, which prevents binding to enzymes that are in the tegument, meaning that these molecules are unable to promote the necessary modifications in the membrane of the parasite tegument leading to the death of the parasite. In the compound 1M, the dihydropiperidinone moiety has been replaced by a trimethoxybenzene ring and the trimethoxybenzene moiety has been replaced by a dicyclohexyl ring. This change in the position of the trimethoxybenzene ring may influence the biological response by causing this compound to lose its activity against the parasite. In compound 6B, the trimethoxybenzene is replaced by a dicyclohexyl ring, as in compound 1M, which may promote the loss of activity against *S. mansoni* parasite when compared with piplartine, because both of these compounds, with this same alteration, lost biological activity.

Complementary studies are still required to determine the relationship between the chemical structure and biological activities of piplartine and its analogues to facilitate the discovery of new prototypes from piplartine. The substitution of a hydrogen atom with a different functional group such as methoxyl, nitro, halogen or others may alter the biological activity, and this may subsequently promote changes in the duration and nature of the pharmacological effect as well as in the physicochemical characteristics of the molecules, thus contributing to the production of new molecules. From this study, it can be observed that functional groups trimethoxybenzene and dihydropiperidinone are important for promoting schistosomicidal activity, because their removal or alteration in modified analogues resulted in the loss of biological activity as well as the toxicity of the compound [26].

In murine fibroblast cells, piplartine showed toxicity at all concentrations tested while compounds 1G and 14B, but not the other derivatives, showed toxicity at 200, 400 and 800 μg/mL. As shown by the solubility parameters (Table 1), ultraviolet and visible light absorption spectra of piplartine and its analogues it was possible to observe that the analogues present values close to that of piplartine, indicating that there are similarities of functional groups between these molecules (Table 1 and Figure 2),; these compounds presented values close to that of piplartine, and the toxicity analysis and bioactivity prediction (Table 2) showed that these synthetic compounds were again similar to piplartine, thus explaining their toxicity. Similarly, flow cytometry cytotoxicity assays in mouse BALB/cN macrophages showed that piplartine was more toxic to mammalian cells than the analogues. However, a marked reduction in the cytotoxicity mediated the by DMSO vehicle was observed in cells treated with piplartine and the analogues, indicating not only that the molecules modulated the toxic effect in mammalian cells but also that they had a protective effect.

Annexin-V FITC/PI staining was used to determine the cell death mechanism observed in the cytotoxicity assays by flow citometry. Recombinant annexin-V conjugated to FITC identifies exposed phosphatidylserine on the inner membrane and is used to detect the initial phase of apoptosis [27], while PI, a red-fluorescent nucleic acid probe to which living cells are impermeable, can be used to detect necrotic cells [28]. In this study, piplartine- and analogue-treated J774A.1 cells showed increased annexin-V FITC staining after 24 h compared to that after PI staining, suggesting that the mechanism of cell death was related to apoptosis [29]. Apoptosis is characterized by cell shrinkage, chromatin condensation and systematic DNA cleavage in a controlled way which prevents the inflammatory process [30]. These data indicate the potential of a rational design of bioactive molecules with low toxicity from piplartine, while further studies are required.

Studies show that the biological and toxicological activity of piplartine can be attributed to the presence of the α, β unsaturated carbonyl groups, where the substitution of any of these unsaturated groups leads a molecule without cytotoxicity and to the loss of biological activity [14,31,32]. Research using this compound has indicated that the α, β unsaturated groups in the aliphatic chain present between the two rings are important for the biological activity against *S. mansoni* as they maintain the conformational arrangement of the two rings, whereas changes in this arrangement decrease cytotoxicity. This effect was observed in the compounds synthesised without the double bond.

This study shows that the modification of piplartine to produce its derivatives resulted in the loss of schistosomicidal activity, indicating that the functional groups trimethoxybenzene and dihydropiperidinone are essential. The synthesis approach used promoted alterations in conformational arrangements, the hydrophilic-lipophilic balance, and the distribution of electronic density compared to those of piplartine. Modifications to trimethoxybenzene and the aliphatic chain were the most significant, as changes to the methoxyl radical in the meta position of the aromatic ring promoted the loss of schistosomicidal activity and subsequent reduction in toxicity.

## 4. Materials and Methods 

### 4.1. Piplartine and Synthetic Analogues

Roots of *Piper tuberculatum* were harvested on the campus of the University of São Paulo (USP), São Paulo, Brazil. Botanical classification was performed by Elsie Franklin Guimarães (Instituto de Pesquisas Jardim Botânico do Rio de Janeiro). A voucher specimen (Kato-0169) has been deposited at the herbarium of the same institute. Piplartine was isolated from the dry roots of *P. tuberculatum* according to a published procedure [25]. Purity was determined by reversed-phase chromatography (RP-HPLC) and the structure by proton nuclear magnetic resonance (1H-NMR) as described also in the supplemental material section (Figure S1).

The cinnamides 1G and 14B were synthesised by adding triethylamine (3 equivalent) and amine (pyrrolidine) to a solution of acid chloride (1 equiv) in CH_2_Cl_2_. To prepare the acid chloride, a solution of 3,4,5-trimethoxycinnamic acid and 3,4-methylenedioxy cinnamic acid (1 equiv.) (Sigma Aldrich, St. Louis, MO, USA) in dry tetrahydrofuran (THF) (10 mL), kept under nitrogen atmosphere, and oxalyl chloride (5 equiv.) was added dropwise and stirred at room temperature for 5–6 h. Excess oxalyl chloride was then removed under reduced pressure, yielding the corresponding acid chloride. The reaction mixture was stirred overnight at room temperature, and quenched with saturated aqueous NH_4_Cl, and extracted with CH_2_Cl_2_ (three times). The combined organic phases were washed with brine and dried over MgSO_4_. After filtration and concentration, the residue was purified by flash chromatography to provide the desired amide [33]. 

Compounds 1M and 6B were synthesised by adding to a solution of 2E-3,4,5-trimethoxycinnamic acid and 4-nitrocinnamic acid (1 equiv.) in THF 0.9 equiv. of *N*,*N′*-dicyclohexylcarbodiimide (DCC). The reaction mixture was stirred overnight at room temperature and dried on a rotary evaporator to remove any remaining solvent. The product was dissolved in (dichloromethane) DCM, a saturated solution of NaHCO_3_ was added, and the resulting solution was washed three times with DCM. The organic phase was combined, dried with MgSO_4_, filtered, and concentrated under pressure. The obtained crystalline product was then recrystallised on hot methanol or purified by chromatography [25,33].

Compound 19A was obtained by adding to a solution of acid chloride (1 equiv.) in THF 1 equiv. butanol. The 2*E*,4*E*-5-3,4,5-trimethoxyphenyl penta-2,4-dienoic acid was synthetised as described previously [34].

### 4.2. Theoretical Calculations

Density Functional Theory (DFT) [35] calculations were performed to determine the energy of the frontier orbitals with b3lyp [36] and basis set 6-311g with two polarisation functions (d,p) and diffuse functions for hydrogen (++), using Gaussian 09 software (Gaussian Inc., Wallingford, CT, USA) for theoretical calculations [37]. Dipole moment structures were calculated to determine electron density using GaussView 5.0 software (Gaussian Inc., Wallingford, CT, USA).

### 4.3. UV–Vis Absorption Spectra

Ultraviolet and visible light (UV–Vis) absorption spectra of piplartine and its analogues were obtained with a UV-1280 spectrophotometer (Shimadzu, Kyoto, Japan). Purified piplartine and its analogues (1G, 1M, 6B and 14B) were solubilised in HPLC-grade acetonitrile and diluted to a concentration of 15 μg/mL. Because of its high absorbance and low solubility, the 19A analogue was further diluted to 7.5 μg/mL before measurement. Scans were performed using a quartz cuvette with a path length of 1 cm in the 200–450 nm range against a baseline recorded with acetonitrile at room temperature.

### 4.4. In Silico Physicochemical and Toxicity Studies 

Two-dimensional structures were designed using Marvin Sketch 17.4.3, 2017, ChemAxon (Available online: http://www.chemaxon.com). 3D structures were geometrically optimised using Another Molecular Modeling Program—Vis (AMMP-Vis) (AMMP 2.0, Monterey, CA, USA), a full-featured molecular dynamics program for manipulating both small molecules and macromolecules including proteins, nucleic acids, and other polymers [38]. Ammp-Mon charges were added and an assisted model building with energy refinement (AMBER) field force was applied to minimise the energy conformation using a genetic algorithm with 3000 steps, applying the water dielectric constant. The conformational search was performed using the Boltzmann jump method, with flexible and psi torsions, at 300 K, and with dielectric constant equal to 80,000 and root mean squared deviation RMSD equal to 60.00. 

Physicochemical properties were also calculated, including molecular weight (MW), lipophilicity (log*_P_*), octanol-water coefficient solubility (logD), water solubility (logS), rotatable bonds, topological polar surface area (tPSA), number of hydrogen bond acceptors and number of hydrogen bond donors using Chemicalize, a web tool to predict basic, geometric, structural and solubility parameters, Calculation, August/2017, (Available online: https://chemicalize.com/; developed by ChemAxon (Available online: http://www.chemaxon.com)). 

The toxicity analyses were performed by pkCSM [39,40]. The biological activity of the parent drug and related compounds was predicted using the Molinspiration web server (http://www.molinspiration.com/).

### 4.5. Cytotoxicity Assay

#### 4.5.1. NIH3T3 and J774A.1 Cell Culture

Murine fibroblast (NIH3T3) cells were acquired from the cell bank of Rio de Janeiro (Rio de Janeiro, Brazil), and the murine BALB/cN macrophage (J774A.1, TIB-67™) (ATCC^®^, Washington, DC, USA). Cells were cultured in Dulbecco’s Modified Eagle’s Medium (DMEM) (Gibco BRL, Grand Island, NY, USA) supplemented with 10% heat inactivated foetal bovine serum (Life, USA) and 1% antibiotic solution (100 IU/mL penicillin/100 µg/mL streptomycin, Life, USA) at 37 °C and 5% CO_2_. 

#### 4.5.2. Cell Viability Assays

Viability assays were performed by the MTT (3-[4,5-dimethylthiazol-2-yl]-2,5-diphenyltetrazolium bromide) dye reduction method. NIH3T3 cells were seeded into 96-well culture plates at a density of 3 × 10^3^ cells in DMEM culture medium overnight at 37 °C and 5% CO_2_ in a humid atmosphere. Next, the medium was removed and various concentrations (25–800 µg/mL) of samples reconstituted in 3% DMSO were applied. DMSO (3%) was used as a control. The plates were incubated for 24 h [28]. Next, 15 µL of MTT solution (5 mg/mL in phosphate buffer) was added to each well and the plates were incubated for 2 h. Medium was discarded and 100 µL of DMSO was added to each well. Absorbance was determined using a spectrophotometer with a microplate reader at 595 nm (Molecular Devices Corporation, Sunnyvale, CA, USA).

For viability assays by flow cytometry method, J774A.1 cells were seeded into 96-well culture plates at a density of 1.0 × 10^5^ cells in DMEM culture medium for 2 h at 37 °C and 5% CO_2_ in a humid atmosphere, and samples reconstituted in 1% DMSO were added to final concentrations of 25, 100 and 400 µg/mL. The plates were incubated for 24 h [28], washed in phosphate buffered saline, and resuspended in 100 µL of binding buffer (10 mM (4-(2-hydroxyethyl)-1-piperazineethanesulfonic acid) HEPES buffer pH 7.4, 140 mM NaCl, and 2.5 mM CaCl_2_). Cells were then treated with 2 µL of annexin-V FITC (Apoptosis Detection Kit; BD Biosciences, Franklin Lakes, NJ, USA) and 2 µL of propidium iodide (PI, 50 µg/mL) for 15 min in the dark at room temperature. Next, 100 µL binding buffer was added and samples were analysed by flow cytometry (BD LSFortessa^TM^, Franklin Lakes, NJ, USA). A total of 20,000 events were collected per sample. 

### 4.6. Animals and Parasites

The Belo Horizonte (BH) strain of *S. mansoni* was maintained in *Biomphalaria glabrata* snails and *Mesocricetus auratus* hamsters at the Adolfo Lutz Institute (São Paulo, Brazil) according to standard procedures. Detailed methods for the infection of molluscs and hamsters, as well as for the recovery of parasites, were as described previously [41]. Briefly, the intermediate host snails were exposed to light for approximately 3 h and cercariae of *S. mansoni* were subsequently harvested. Hamsters (aged 3 weeks, weight 25 g) were infected subcutaneously with 150 cercariae and, 49 days post-infection, adult *S. mansoni* specimens were recovered from each hamster by perfusion with Roswell Park Memorial Institute (RPMI) medium 1640 medium (Invitrogen, São Paulo, Brazil) and heparin. This study was approved by the Institutional Review Board of the Guarulhos University (São Paulo, Brazil; approval number 31/17, 20 March 2017). All animals were handled in strict accordance with good animal practice as defined by the Animal Use Ethics Committee.

### 4.7. Adult Schistosomes

The preparations and culture of schistosomes was performed as previously described [42,43]. Briefly, hamsters were killed 49 days after infection for the recovery of adult schistosomes. Next, fresh adult worm pairs (male and female) were washed and placed in a 24-well culture plate (TPP, St. Louis, MO, USA) containing RPMI 1640 medium supplemented with 10% foetal bovine serum (Gibco-BRL, Life Technologies do Brazil Ltda, Sao Paulo, SP, Brazil), 200 µg/mL streptomycin, and 200 IU/mL penicillin (Invitrogen, São Paulo, SP, Brazil) at 37 °C in a 5% CO_2_ atmosphere. Adult schistosomes were maintained continuously in medium (with or without drugs) for 96 h as described below.

### 4.8. In Vitro Experiments with Adult Worms of S. mansoni

In vitro assays in *S. mansoni* adults were performed with mated males and females. The worm pairs obtained from the infusion hamsters were washed twice with RPMI 1640 medium containing 200 U/mL penicillin, 200 μg/mL streptomycin and 2 μg/mL amphotericin B (Vitrocell, Campinas, Brazil). The mated parasites were then transferred into 24-well cell culture plates (TPP, St. Louis, MO, USA) containing, per well, 1 pair of worms in 2 mL of RPMI 1640 medium supplemented with 10% foetal bovine serum and buffered with 25 mM HEPES. Worms were incubated with the amide pyramethrin analyzes at different concentrations (50, 25, 10 and 5 μM); 5 and 10 μM piplartine for up to 96 h; 2 μM praziquantel was used as positive control and culture medium was used as a negative control [44]. 

Cultures of adult worms were monitored by microscopy or magnifying glass. The effect of the drug was assessed with emphasis on changes in worm motor activity and death of worms as previously described [45,46,47]. The mortality of worms was judged by the absence of movement for 2 min or when touched with forceps. 

### 4.9. Statistical Analysis

Statistical analysis was performed by analysis of variance (ANOVA) and Bonferroni post hoc test using Graph Pad Prism 5.03 (GraphPad Software, La Jolla, CA, USA) to evaluate the statistical significance of differences between control and treated cells in cytotoxicity assays. All values were expressed as means ± standard error of the mean (SEM) from triplicates in three independent experiments, and a value of *p* < 0.05 was considered statistically significant.

## 5. Conclusions

This study demonstrated that analogues of piplartine, when compared with the parent molecule, showed a loss of activity against the *S. mansoni* parasite, but showed decreased toxicity compared to that of piplartine. Alterations in the trimethoxybenzene ring of piplartine, following unsaturation of the aliphatic chain and the dihydropiperidinone ring, were shown to be highly sensitive to modification, promoting the loss of biological activity and decreased toxicity. These findings indicate that analogous molecules within the same series do not present the same physicochemical and biological activity.

## Figures and Tables

**Table 1:** Basic, geometric, structural, and solubility parameters of the parent and analogue compounds.

Compounds	Piplartine	19A	14B	6B	1M	1G
**Basic properties**						
Atom count	40	51	31	48	56	38
Mw	315.32	287.31	432.48	387.39	239.27	359.42
**Structural properties**						
Asymmetric atom count	0	0	0	0	0	0
Rotatable bond count	5	6	2	5	7	5
Ring count	2	2	3	3	3	2
Aromatic ring count	2	1	2	3	3	2
Hetero ring count	1	1	2	0	0	1
Hydrogen bond donor count	0	0	0	3	1	0
Hydrogen bond acceptor count	5	5	2	5	5	4
Formal charge	0	0	0	0	0	0
Fsp3 (a)	0.18	0.40	0.13	0.00	0.12	0.19
Topological polar surface area (A^2^)	65.07	65.07	31.23	93.11	77.10	49.69
Molar refractivity cm^3^/mol	87.42	101.15	70.63	112.82	123.64	79.89
Polarisability (A^3^)	32.54	38.29	26.66	41.46	46.66	30.59
**Solubility parameters**						
log*_P_*	1.89	2.71	2.36	3.55	4.63	2.26
Milog*p*(b)	2.19	2.78	4.69	5.02	2.86	2.90
log*_D_* pH range 1.7–8.0	1.89	2.71	2.36	3.54	4.63	2.26
Intrinsic solubility	−3.01	−4.53	−2.87	−4.73	−5.48	−2.81
Solubility category	High	Moderate	High	Low	Low	High
logS pH range 1.7–8.0	−3.01	−4.53	−2.87	−4.73	−5.48	−2.81
**Geometry parameters**						
Van der Waals volume (Å^3^)	281.06	337.61	213.99	341.62	390.54	260.80
Volume (Å^3^) (b)	282.84	263.86	394.07	340.76	218.97	339.43
Van der Waals surface area (Å^2^)	425.62	543.27	333.75	515.78	620.14	423.69
Solvent accessible surface area (Å^2^)	549.15	659.24	445.82	631.17	725.57	577.96
Topological polar surface area (Å^2^)	65.07	65.07	31.2	93.11	77.10	49.69
Absorbance	322 nm	329 nm	321 nm	312 nm	306.5 nm	301.5 nm

(a) Number of sp3 carbons/number of carbons; (b) Molinspiration method. 19A, 14B, 6B, 1M, and 1G are analogue compounds of piplartine. Fraction of sp^3^ carbon atoms (Fsp^3^).

**Table 2:** Toxicity analysis and bioactivity prediction of the parent and analogue compounds against various drug targets.

Compounds	Piplartine	19A	14B	6B	1M	1G
**Toxicity prediction (c)**									
AMES toxicity	Yes	No	No	No	No	No
Max. tolerated dose (human) (0.505 log mg/kg/day)	1.006	0.677	1.043	1.095	1.051	1.114
hERG I inhibitor	No	No	No	No	No	No
hERG II inhibitor	No	No	No	Yes	Yes	No
Oral Rat Acute Toxicity (LD_50_) (2.661 mol/kg)	2.268	2.295	2.413	2.195	2.37	2.389
Oral Rat Chronic Toxicity (LOAEL) (3.402 log mg/kg bw/day)	1.64	1.787	1.812	2.455	2.388	1.592
Hepatotoxicity	No	Yes	No	Yes	Yes	No
Skin sensitisation	No	No	No	No	No	No
*T. pyriformis* toxicity (0.285 × log µg/L)	1.138	1.359	2.004	0.437	0.658	1.591
Minnow toxicity (5.577 × log mM)	1.293	0.446	1.179	−0.172	−0.914	1.027
**Predictive bioactivity (b)**									
GPCR ligand	0.13	0.17	0.07	−0.11	−0.04	−0.15
Ion channel modulator	−0.51	−0.16	−0.53	−0.27	−0.29	−0.54
Kinase inhibitor	−0.13	−0.12	−0.13	−0.20	−0.09	−0.14
Nuclear receptor ligand	−0.32	−0.07	−0.12	−0.31	−0.30	−0.31
Protease inhibitor	−0.40	0.02	−0.39	−0.38	−0.33	−0.48
Enzyme inhibitor	−0.02	0.05	0.24	−0.17	−0.12	−0.00

(a) Number of *sp3* carbons/number of carbons; (b) Molinspiration method; (c) pkCSM. The Ames test is a widely employed method that uses bacteria to test whether a given chemical can cause mutations in the DNA of the test organism (Ames); The human ether-a-go-go-related gene (hERG); median lethal dose (LD_50_); Lowest-Observed-Adverse-Effect Level (LOAEL); G-protein-coupled receptors (GPCR).

**Table 3:** Determination of pD_2_ and E_max_ for the cytotoxic effect of piplartine and its analogues in murine fibroblast (NIH-3T3) cells.

Compounds	pD_2_ (−logIC_50_) ^a^	E_max_ (%) ^b^
19A	−3.06 ± 0.15	37.93 ± 2.85
1G	−2.00 ± 0.03	95.69 ± 0.79
1M	−2.31 ± 0.04	70.94 ± 1.20
14B	−1.95 ± 0.04	83.64 ± 0.47
6B	−2.80 ± 0.08	47.93 ± 2.52
Piplartine	5.68 ± 2.95	96.94 ± 0.12

^a^ Negative logarithm of the mean inhibitory concentration (IC_50_); ^b^ E_max_: decrease in cell viability at the maximum concentration tested (800 μg/mL).

**Table 4:** In vitro effects of compounds in 49-day-old *S. mansoni* worms.

Group	Period of Incubation (h)	Dead Worms (%) ^a^		Motor Activity Reduction (%) ^a^			
				Slight		Significant	
		M	F	M	F	M	F
Control ^b^	24	0	0	0	0	0	0
	48	0	0	0	0	0	0
	72	0	0	0	0	0	0
	96	0	0	0	0	0	0
0.5% DMSO	24	0	0	0	0	0	0
	48	0	0	0	0	0	0
	72	0	0	0	0	0	0
	96	0	0	0	0	0	0
Praziquantel	24	100	100	0	0	100	100
2 µM	48	100	100	0	0	100	100
	72	100	100	0	0	100	100
	96	100	100	0	0	100	100
Amide piplartine	24	100	100	0	0	100	100
10 µM	48	100	100	0	0	100	100
5 µM	72	100	100	0	0	100	100
	96	100	100	0	0	100	100
	24	0	0	100	100	0	0
	48	0	0	100	100	0	0
	72	0	0	100	100	0	0
	96	60	60	0	0	60	60

^a^ Percentages relative to the 20 worms investigated; ^b^ RPMI 1640. The effect of the compounds on motor activity of adult *S. mansoni* was assessed qualitatively. Male (M) and Female (F).

## Supplementary Materials

Supplementary materials can be found at http://www.mdpi.com/1422-0067/19/6/1802/s1.

## Abbreviations

DMSO	Dimethyl sulfoxide
THF	Tetrahydrofuran
DCC	Dicyclohexylcarbodiimide
