# Supplementary material for: Structure–Activity Relationship of Piplartine and Synthetic Analogues against Schistosoma mansoni and Cytotoxicity to Mammalian Cells
Source: Int J Mol Sci. 2018 Jun 19;19(6):1802. doi: 10.3390/ijms19061802 (PMC6032158; doi:10.3390/ijms19061802)
Supplement: Supplementary file 1 [file ijms-19-01802-s001.pdf]

Supplementary Materials

Table S1. *In vitro* effect of piplartine and analogues against 49-day-old *S. mansoni*.

Group	Period of incubation (h)	Dead worms (%)[a]		Motor activity reduction (%)[a]			
				Slight		Significant	
		M	F	M	F	M	F
Control [b]	24	0	0	0	0	0	0
	48	0	0	0	0	0	0
	72	0	0	0	0	0	0
	96	0	0	0	0	0	0
0.5% DMSO	24	0	0	0	0	0	0
	48	0	0	0	0	0	0
	72	0	0	0	0	0	0
	96	0	0	0	0	0	0
Praziquantel	24	100	100	0	0	100	100
2 µM	48	100	100	0	0	100	100
	72	100	100	0	0	100	100
	96	100	100	0	0	100	100
**Amide piplartine**	24	100	100	0	0	100	100
10 µM	48	100	100	0	0	100	100
	72	100	100	0	0	100	100
	96	100	100	0	0	100	100
5 µM	24	0	0	100	100	0	0
	48	0	0	100	100	0	0
	72	0	0	100	100	0	0
	96	60	60	0	0	60	60
**Amide 1G**	24	0	0	0	0	0	0
50 µM	48	0	0	0	0	0	0
	72	0	0	0	0	0	0
	96	0	0	0	0	0	0
25 µM	24	0	0	0	0	0	0
	48	0	0	0	0	0	0
	72	0	0	0	0	0	0

	96	0	0	0	0	0	0
10 µM	24	0	0	0	0	0	0
	48	0	0	0	0	0	0
	72	0	0	0	0	0	0
	96	0	0	0	0	0	0
5 µM	24	0	0	0	0	0	0
	48	0	0	0	0	0	0
	72	0	0	0	0	0	0
	96	0	0	0	0	0	0
**Amide 1M**	24	0	0	0	0	0	0
50 µM	48	0	0	0	0	0	0
	72	0	0	0	0	0	0
	96	0	0	0	0	0	0
25 µM	24	0	0	0	0	0	0
	48	0	0	0	0	0	0
	72	0	0	0	0	0	0
	96	0	0	0	0	0	0
10 µM	24	0	0	0	0	0	0
	48	0	0	0	0	0	0
	72	0	0	0	0	0	0
	96	0	0	0	0	0	0
5 µM	24	0	0	0	0	0	0
	48	0	0	0	0	0	0
	72	0	0	0	0	0	0
	96	0	0	0	0	0	0
**Amide 6B**	24	0	0	0	0	0	0
50 µM	48	0	0	0	0	0	0
	72	0	0	0	0	0	0
	96	0	0	0	0	0	0
25 µM	24	0	0	0	0	0	0
	48	0	0	0	0	0	0
	72	0	0	0	0	0	0
	96	0	0	0	0	0	0

25 µM	24	0	0	0	0	0	0
	48	0	0	0	0	0	0
	72	0	0	0	0	0	0
	96	0	0	0	0	0	0
10 µM	24	0	0	0	0	0	0
	48	0	0	0	0	0	0
	72	0	0	0	0	0	0
	96	0	0	0	0	0	0
5 µM	14	0	0	0	0	0	0
	48	0	0	0	0	0	0
	72	0	0	0	0	0	0
	96	0	0	0	0	0	0
**Amide 14B**	24	0	0	0	0	0	0
50 µM	48	0	0	0	0	0	0
	72	0	0	0	0	0	0
	96	0	0	0	0	0	0
25 µM	24	0	0	0	0	0	0
	48	0	0	0	0	0	0
	72	0	0	0	0	0	0
	96	0	0	0	0	0	0
10 µM	24	0	0	0	0	0	0
	48	0	0	0	0	0	0
	72	0	0	0	0	0	0
	96	0	0	0	0	0	0
5 µM	24	0	0	0	0	0	0
	48	0	0	0	0	0	0
	72	0	0	0	0	0	0
	96	0	0	0	0	0	0
**Amide 19A**	24	0	0	0	0	0	0
50 µM	48	0	0	0	0	0	0
	72	0	0	0	0	0	0
	96	0	0	0	0	0	0
25 µM	24	0	0	0	0	0	0

	48	0	0	0	0	0	0
	72	0	0	0	0	0	0
	96	0	0	0	0	0	0
10 μM	24	0	0	0	0	0	0
	48	0	0	0	0	0	0
	72	0	0	0	0	0	0
	96	0	0	0	0	0	0
5 μM	24	0	0	0	0	0	0
	48	0	0	0	0	0	0
	72	0	0	0	0	0	0
	96	0	0	0	0	0	0

[a] Percentages relative to the 20 worms investigated.
[b] RPMI 1640.
The effect of the compounds on motor activity of adult *S. mansoni* was assessed qualitatively.

Figure S1. Description of piplartine and its analogues.

Piplartine, IUPAC name:

1-[(2E)-3-(3,4,5-trimethoxyphenyl)prop-2-enoyl]-1,2,5,6-tetrahydropyridin-2-one

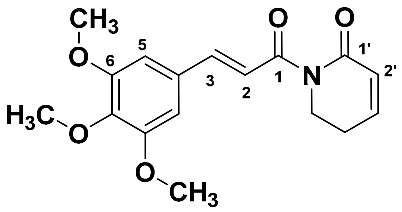

RMN^1 H (300 MHz; $CDCl_3$): δ 7,68 (d; *J*=15,6 Hz; 1H, H2) ; 7,43 (d; *J*=15,6 Hz; 1H, H3); 6,95 (m, 1H, H2'); 6,81 (s, 2H, H5 e H9); 6,04 (dt; *J*=9,9 e 1,8 Hz; 1H; H3'); 4,05 (t; *J*=6,5 Hz; 2H; H5'); 3,89 (s, 6H; OMe 6 e 8); 3,88 (s; 3H; OMe 7); 2,50 (m; 2H, H4').
RMN ^{13}C (75 MHz; $CDCl_3$): δ 168,70 (C1); 165,80 (C1'); 153,30 (C6, C8); 145,50 (C3'); 143,70 (C4); 139,90 (C7); 130,60 (C3); 125,70 (C2'); 121,00 (C2); 105,40 (C5, C9); 60,89 (OMe 7); 56,11 (OMe 6 e 8); 41,62 (C5'); 24,70 (C4').
EMBR- *m/z* (%): 317 (M^+, 90), 289 (20), 274 (32), 221 (100), 205 (20), 190 (32), 177 (17).
EMAR (IES): calculated to $C_{17}H_{20}NO_5^+[M+H]^+$ = 318,1336; found = 318,1337.

1G, IUPAC name:

(2E)-1-(pyrrolidin-1-yl)-3-(3,4,5-trimethoxyphenyl)prop-2-en-1-one

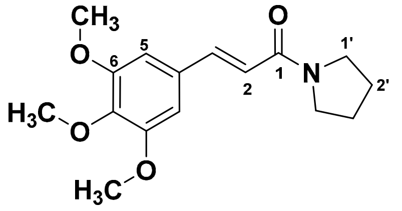

RMN ^{1}H (300 MHz; $CDCl_3$): δ 7,62 (d; *J*=15,3 Hz; 1H; H3); 6,75 (s, 2H, H5, H9); 6,62 (d; *J*=15,3 Hz; 1H; H2); 3.89 (s, 6H; OMe 6 e 8); 3,87 (s, 3H, OMe 7); 3,65-3,60 (m, 4H, H1', H4'); 2,01 (m, 2H, H2'); 1,91 (m, 2H, H3').

RMN ^{13}C (75 MHz; $CDCl_3$): δ 164,52 (C1); 153,25 (C6, C8); 141,64 (C3); 139,40 (C7); 130,80 (C4); 117,98 (C2); 104,95 (C5, C9); 60,82 (OMe 7); 56,07 (OMe 6 e 8); 46,51 (C1'); 45,96 (C4'); 26,02 (C2'); 24,23 (C3').

EMAR (IES): calculated to $C_{16}H_{22}NO_4^+$[M+H]$^+$ = 292,1543; found = 292,1539 .

1M, IUPAC name:

1,3-dicyclohexyl-1-[(2E)-3-(3,4,5-trimethoxyphenyl)prop-2-enoyl]urea

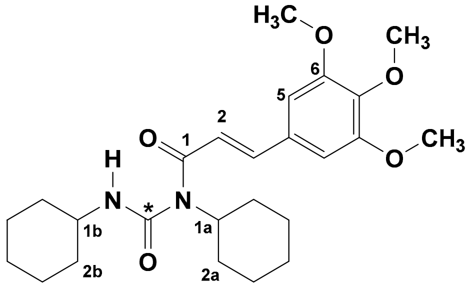

RMN ^{1}H (500 MHz; $CDCl_3$): δ 7,57 (d; *J*=15,5 Hz; 1H; H2); 6,70 (s, 2H; H5, H9); 6,62 (d; *J*=15,5 Hz; 1H; H3); 4,16 (m, 1H, H1b); 3,88 (s, 6H; OMe 6 e 8); 3,86 (s, 3H, OMe 7), 3,76 (m, 1H, H1a); 2,02–1,82 (m, 8H, H2a, H2b, H6a, H6b), 1,41–1,15 (m, 12H; H3a, H3b, H4a, H4b, H5a, H5b).

RMN ^{13}C (100 MHz; $CDCl_3$): δ 165,89 (C1); 154,05 (C*); 153,35 (C6, C8); 143,08 (C3); 139,88 (C7); 130,18 (C4); 118,64 (C2); 105,02 (C5, C9); 60,90 (OMe 7); 55,99 (OMe 6 e 8); 55,49 (C1b); 49,98 (C1a); 34,87 (C2b); 33,90 (C6b); 32,77 (C2a); 30,86 (C6a); 26,15 (C3b, C5b); 25,37 (C3a); 25,34 (C5a); 24,67 (C4a, C4b).

EMBR-*m/z* (%): 319 (40), 236 (100), 222 (55), 221 (50), 207 (32), 194 (25), 190 (30), 180 (27), 163 (20), 133 (21), 121 (20), 98 (45), 77 (20), 68 (20), 55 (25).

EMAR (IES): calculated to $C_{25}H_{37}N_2O_5^+$ [M+H]$^+$ = 445,2697; found = 445,2725.

IV (KBr, $\nu_{máx}$/cm^{-1}): 3291, 2997, 2931, 2854, 2249, 2118, 1761, 1703, 1650, 1583, 1330, 1129, 1006, 973, 892, 822.

6B, IUPAC name:

1,3-dicyclohexyl-1-[(2E)-3-(4-nitrophenyl)prop-2-enoyl]urea

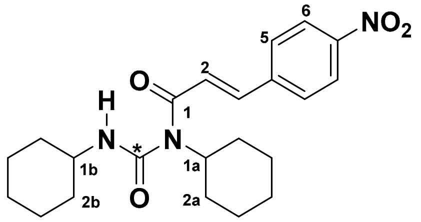

RMN ^{1}H (300 MHz; $CDCl_3$): δ 8,24 (d; *J*=9,0 e 2,4 Hz; 2H, H6, H8); 7,69 (d; *J*=15,3 Hz; 1H, H2); 7,62 (d; *J*=9,0 e 2,1 Hz; 2H, H5, H9); 6,84 (d; *J*=15,3 Hz; 1H, H3); 6,55 (sl, NH); 4,19–4,11 (m, 1H, H1b); 3,82–3,74 (m, 1H, H1a); 1,89–1,19 (m, 20H, H2a, H2b, H3a, H3b, H4a, H4b, H5a, H5b, H6a, H6b).

RMN ^{13}C (75 MHz; $CDCl_3$): δ 165,16 (C1); 153,66 (C*); 148,28 (C7); 140,92 (C4); 140,23 (C3); 128,41(C5, C9); 124,19 (C6, C8); 123,61 (C2); 55,73 (C1a); 50,05 (C1b); 34,89 (C2a, C6a); 32,78 (C2b); 31,00 (C6b); 26,16 (C3a); 25,42 (C3b); 25,35 (C4a); 25,30 (C4b); 24,66 (C5a, C5b).

EMAR (IES): calculated to $C_{22}H_{30}N_3O_4$+: $[M+H]^+$ = 400,2231; found = 400,2236.

14B, IUPAC name:

(2E)-3-(2H-1,3-benzodioxol-5-yl)-1-(pyrrolidin-1-yl)prop-2-en-1-one

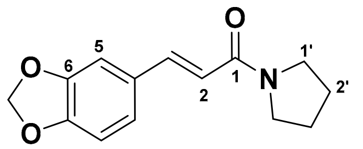

RMN ^{1}H (300 MHz; $CDCl_3$): δ 7,61 (d; *J*=15,3 Hz; 1H, H3); 7,04 (d; *J*=1,5 Hz; 1H, H5); 7,02-6,99 (dd; *J*=8,4 e 1,8 Hz; 1H, H9); 6,80 (d; *J*=8,1 Hz; 1H, H8); 6,56 (d; *J*=15,3 Hz; 1H, H2); 5,99 (s, 2H, OCH_2O); 3,63–3,56 (m, 4H, H1’, H4’) 2,04 -1,98 (m; 2H, H2’); 1,94–1,87 (m, 2H, H3’).

RMN ^{13}C (75 MHz; $CDCl_3$): δ 164,76 (C1); 148,84 (C6); 148,10 (C7); 141,30 (C3); 129,68 (C4); 123,75 (C9); 116,82 (C2); 108,41 (C8); 106,32 (C5); 101,32 (OCH_2O); 46,47 (C1’); 45,95 (C4’); 26,07 (C2’); 24,28 (C3’).

EMAR (IES): calculated to $C_{14}H_{16}NO_3^+$$[M+H]^+$ = 246,1125; found = 246,1130.

19A IUPAC name:

Butyl (2E,4E)-5-(3,4,5-trimethoxyphenyl)penta-2,4-dienoate

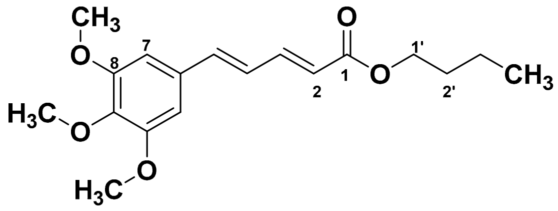

RMN [1]H (300 MHz; $CDCl_3$): δ 7,42 (qd; *J*=15,5 e 8,9 Hz; 1H, H3); 6,81 (sl, 1H, H5); 6,78 (sl, 1H, H4); 6,69 (s, 2H, H7, H11); 5,99 (d; *J*=15,3 Hz; 1H, H2); 4,18 (*t*, *J*=6,8 Hz; 2H, H1'); 3,90 (s, 6H; OMe 8, 10); 3,87 (s, 3H; OMe 9); 1,72-1,63 (m, 2H, H2'); 1,49–1,39 (m, 2H, H3'); 0,96 (t, *J*=7,4 Hz; 3H, H4').

RMN [13]C (75 MHz; $CDCl_3$): δ 167,11 (C1); 153,37 (C8, C10); 144,30 (C3); 140,21 (C5); 139,10 (C9); 131,62 (C2); 125,64 (C6); 121,03 (C4); 104,26 (C7, C11); 64,22 (C1'); 60,91 (OMe 9); 56,10 (OMe 8, 10); 30,73 (C2'); 19,15 (C3'); 13,70 (C4').

EMBR-*m/z* (%): 320 (25), 219 (100), 204 (30), 188 (55).

EMAR (IES): calculated to $C_{18}H_{24}NaO^{+}[M+Na]^{+}$ = 343,1516; found = 343,1513.

IV (KBr, v_{max}/cm^{-1}): 3445, 3005, 2960, 2897, 2838, 1703, 1624, 1579, 1506, 1467, 1419, 1357, 1253, 1244, 1142, 998, 850, 744, 724.

Figure S2. Original data from mouse BALB/cN macrophage (J774A.1) cell line analyzed by flow cytometry (10.000 events/sample) to evaluate the cell death mechanism mediated by piplartine and analogues 1G 1M, 6B, 14B and 19A, following treatment for 24 h at 25, 100 and 400 µg/mL concentrations using Annexin-V FITC (apoptosis marker) and Propidium Iodide (PI, necrosis marker) staining. The controls DMEM, DMSO and H_2O_2 (peroxido) were used as comparisons parameters.

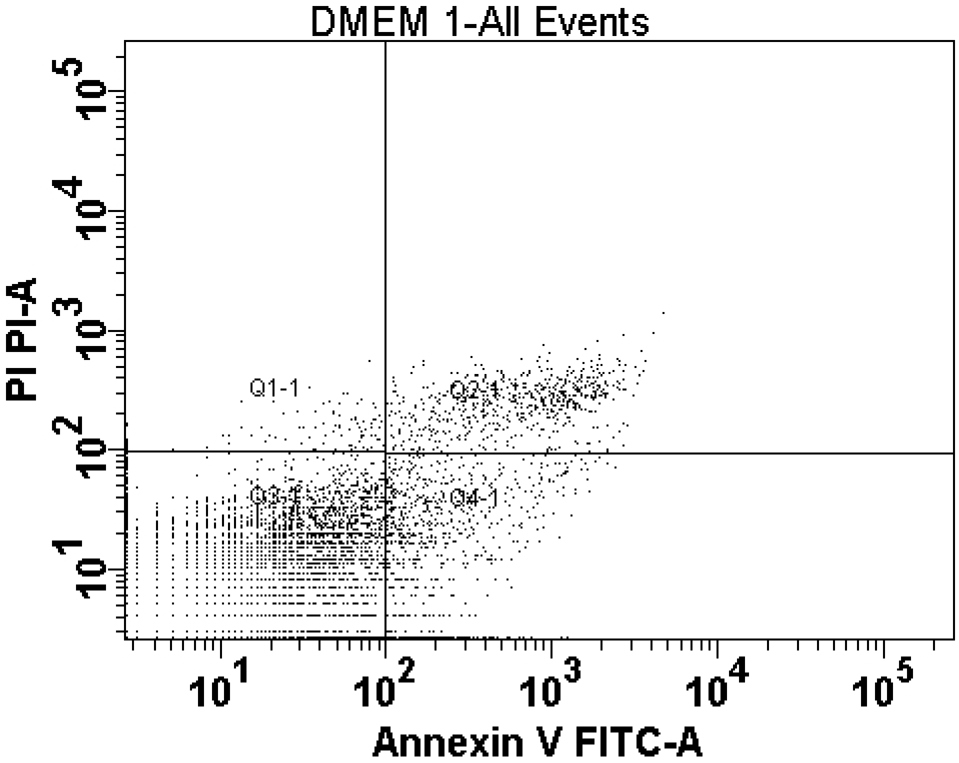

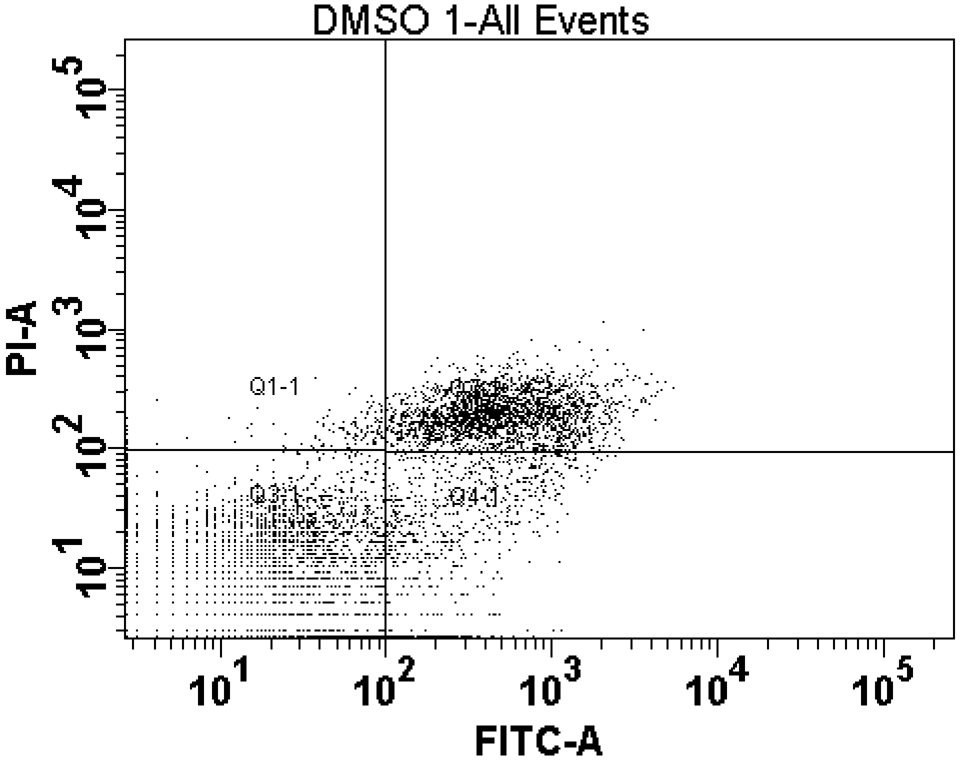
DMSO 1-All Events
PI-A
FITC-A
Q1-1
Q3-1
Q4-1
10^1
10^2
10^3
10^4
10^5

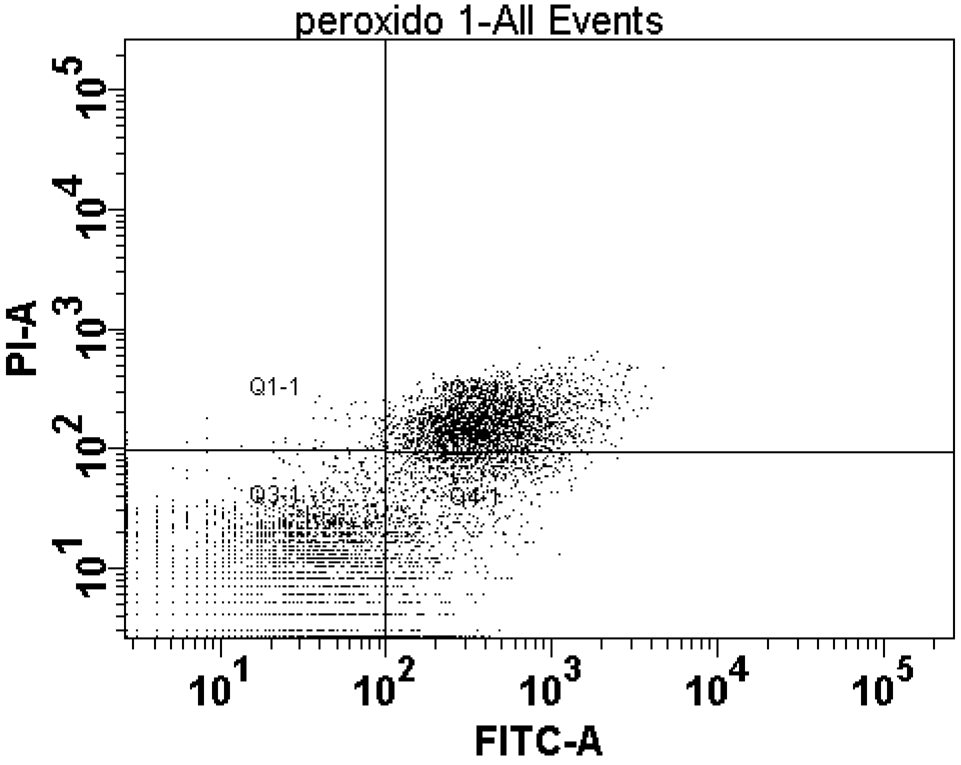
peroxido 1-All Events
PI-A
FITC-A
Q1-1
Q3-1
Q4-1
10^1
10^2
10^3
10^4
10^5

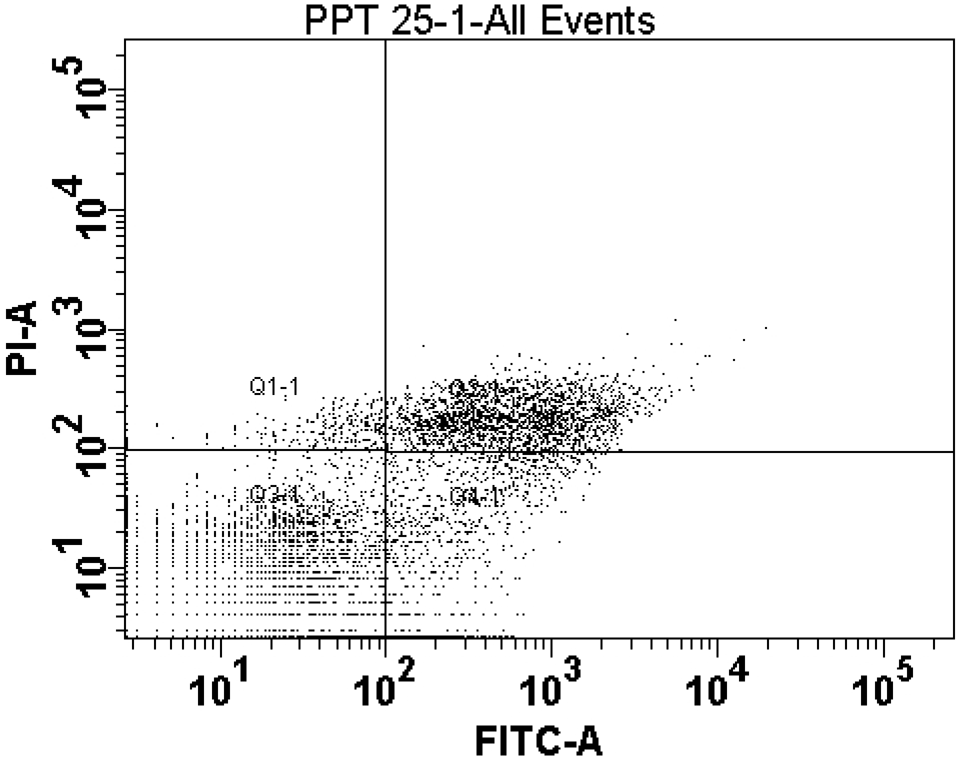
PPT 25-1-All Events
PI-A
FITC-A
Q1-1
Q2-1
Q3-1
Q4-1
10^1
10^2
10^3
10^4
10^5

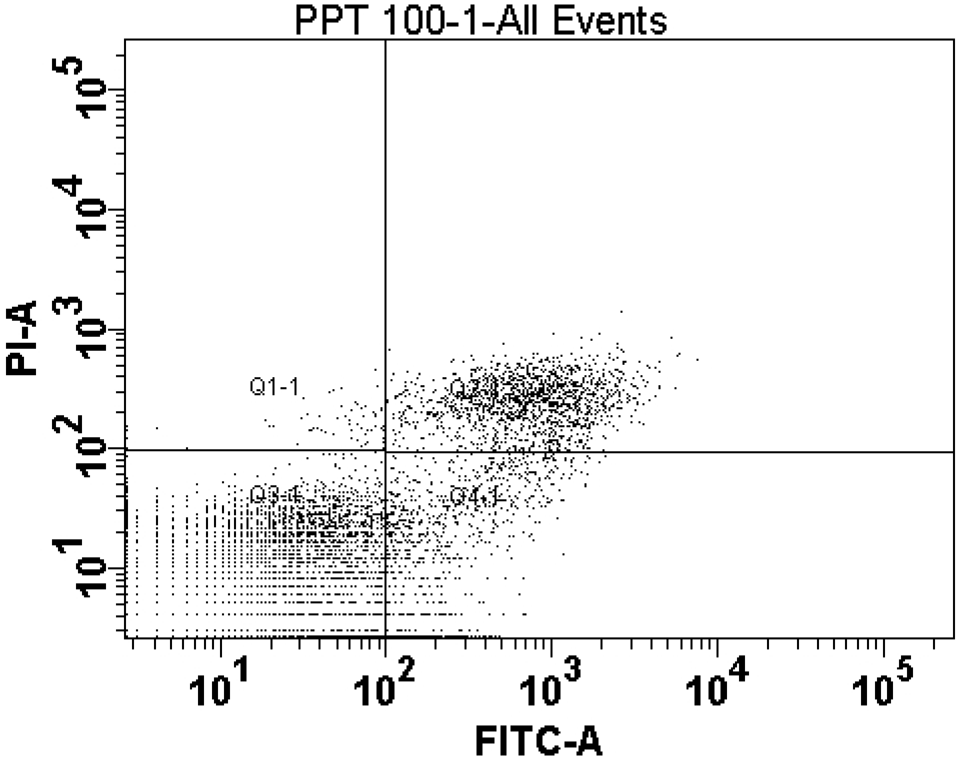
PPT 100-1-All Events
PI-A
FITC-A
Q1-1
Q2-1
Q3-1
Q4-1
10^1
10^2
10^3
10^4
10^5

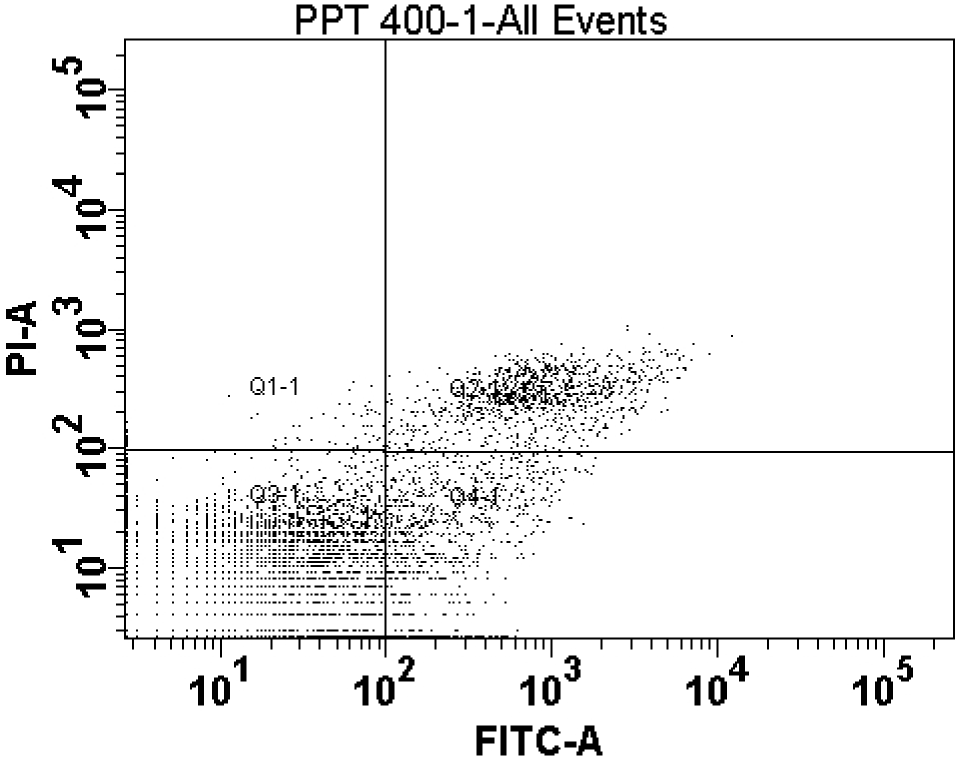
PPT 400-1-All Events
Q1-1
Q2-1
Q3-1
Q4-1
PI-A
FITC-A
10^1
10^2
10^3
10^4
10^5

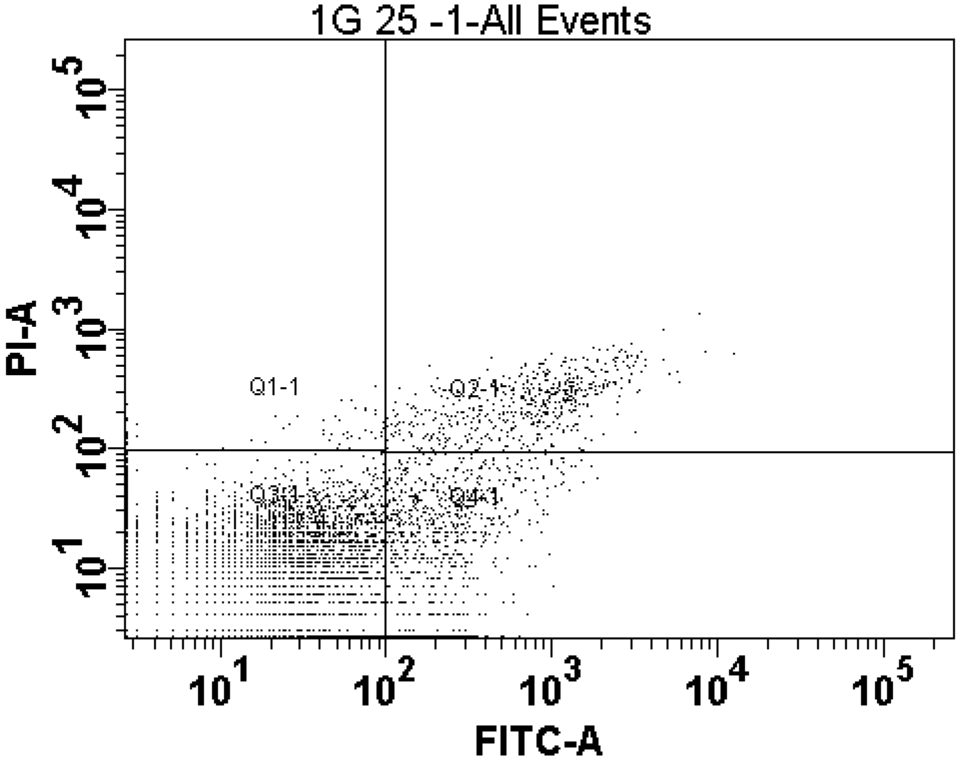
1G 25 -1-All Events
Q1-1
Q2-1
Q3-1
Q4-1
PI-A
FITC-A
10^1
10^2
10^3
10^4
10^5

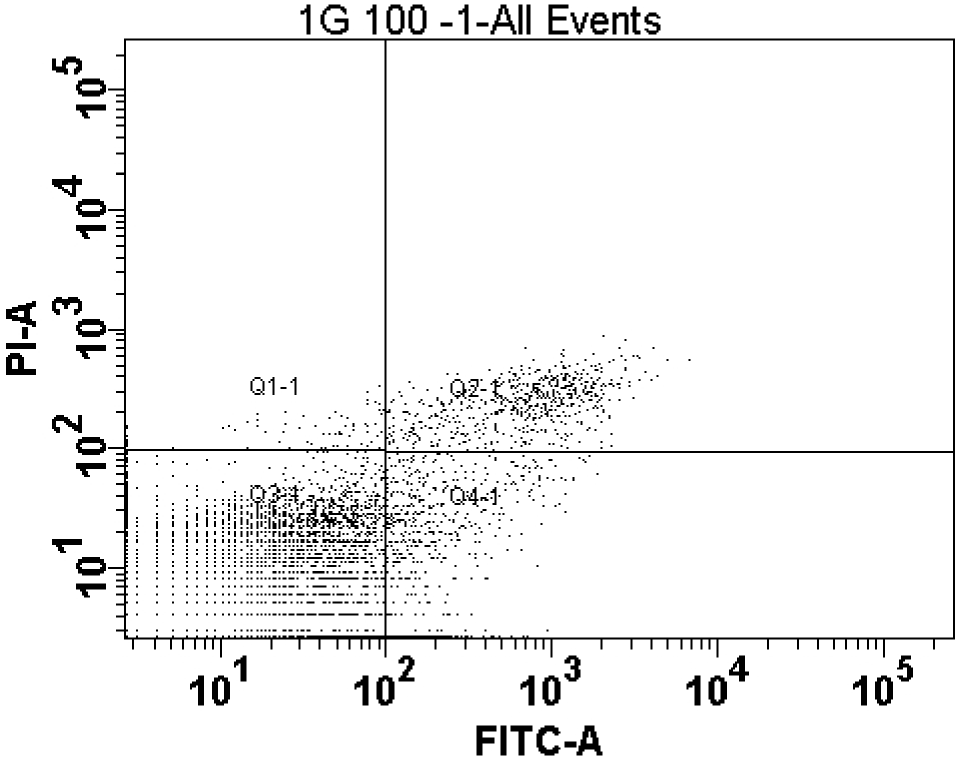
1G 100 -1-All Events
PI-A
FITC-A
Q1-1
Q2-1
Q3-1
Q4-1
10^1
10^2
10^3
10^4
10^5

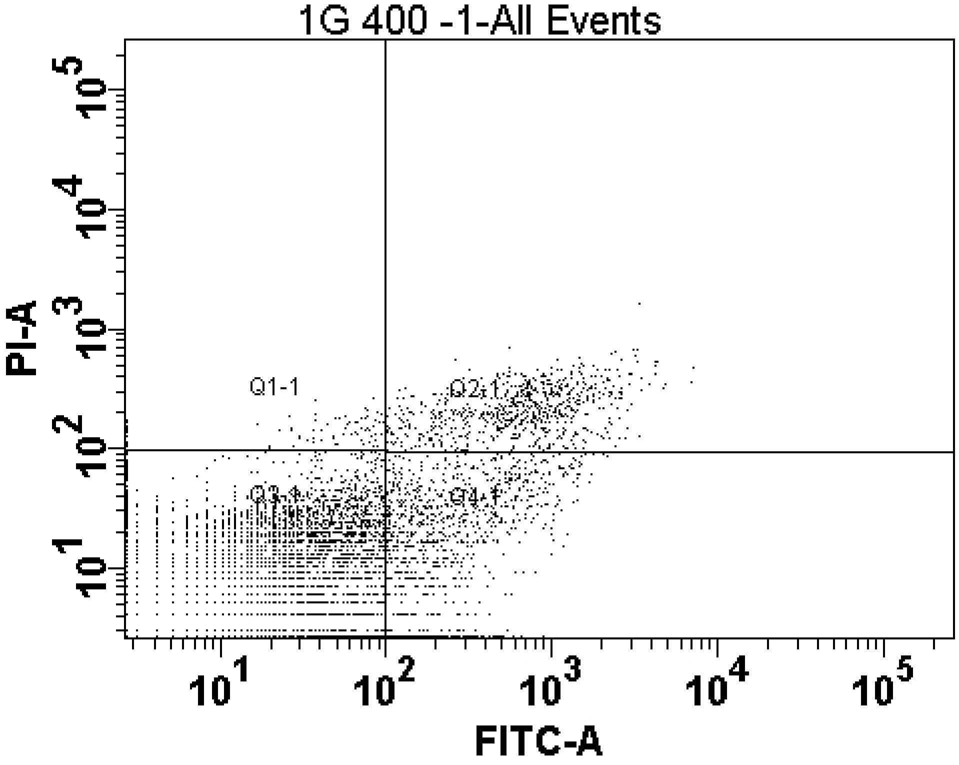
1G 400 -1-All Events
PI-A
FITC-A
Q1-1
Q2-1
Q3-1
Q4-1
10^1
10^2
10^3
10^4
10^5

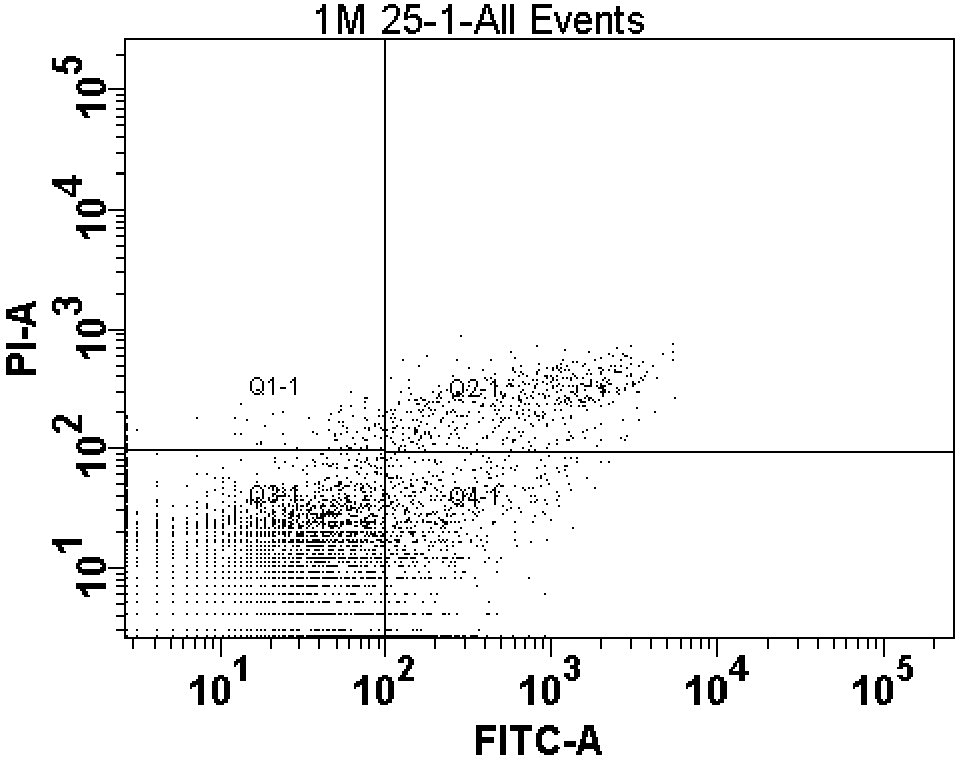
1M 25-1-All Events
PI-A
FITC-A
Q1-1
Q2-1
Q3-1
Q4-1

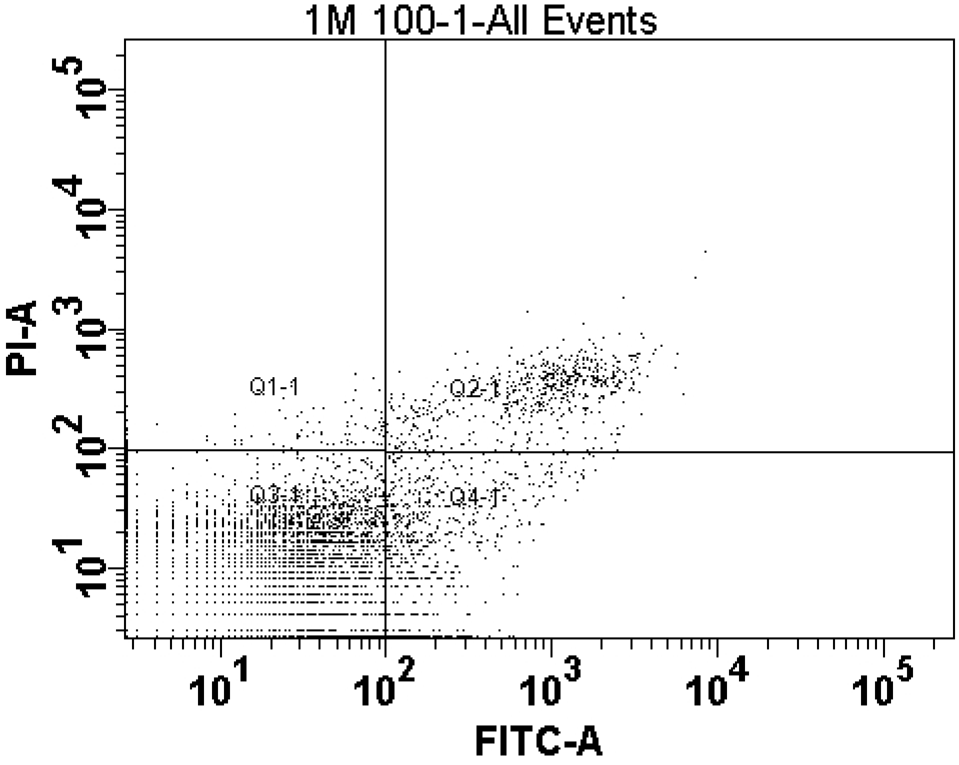
1M 100-1-All Events
PI-A
FITC-A
Q1-1
Q2-1
Q3-1
Q4-1

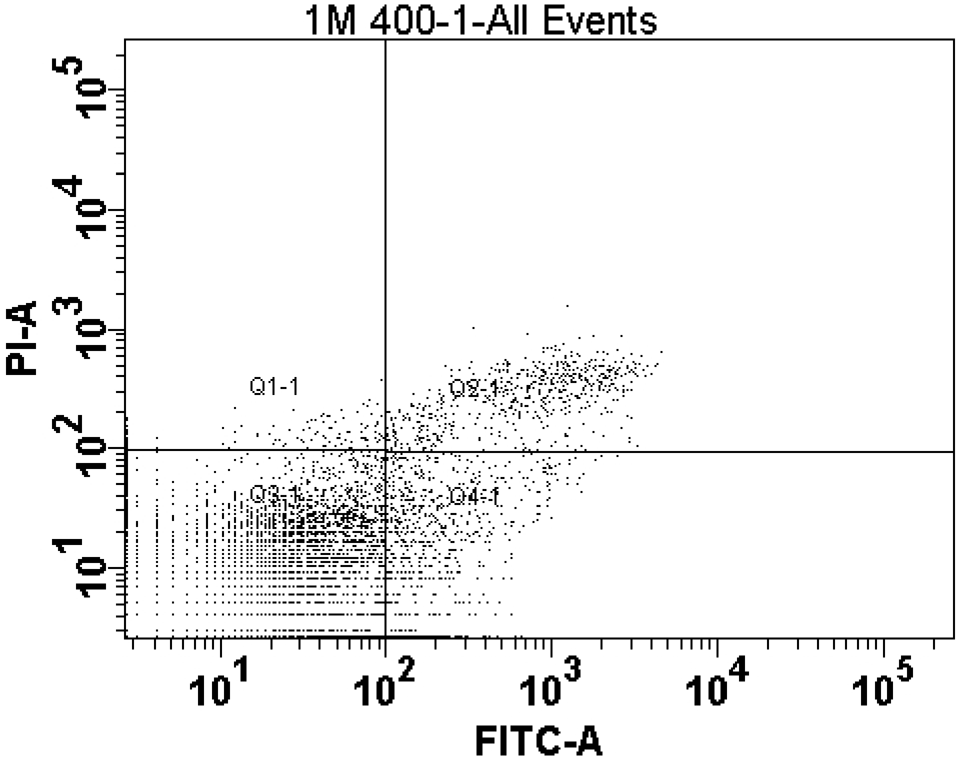
1M 400-1-All Events
Q1-1
Q2-1
Q3-1
Q4-1
PI-A
FITC-A

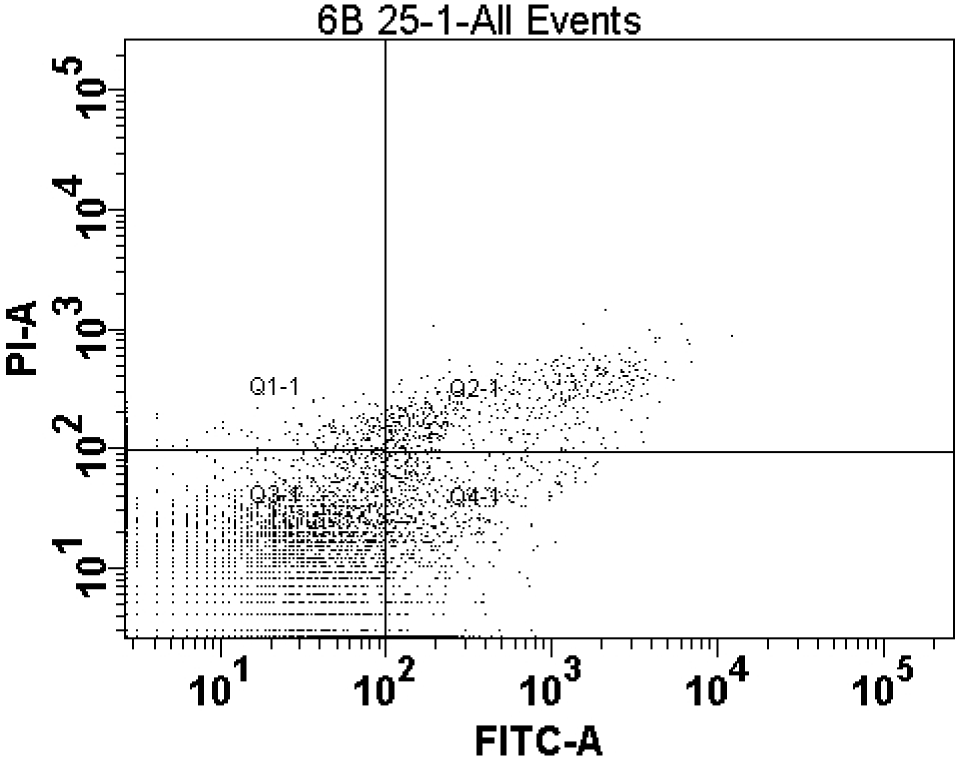
6B 25-1-All Events
Q1-1
Q2-1
Q3-1
Q4-1
PI-A
FITC-A

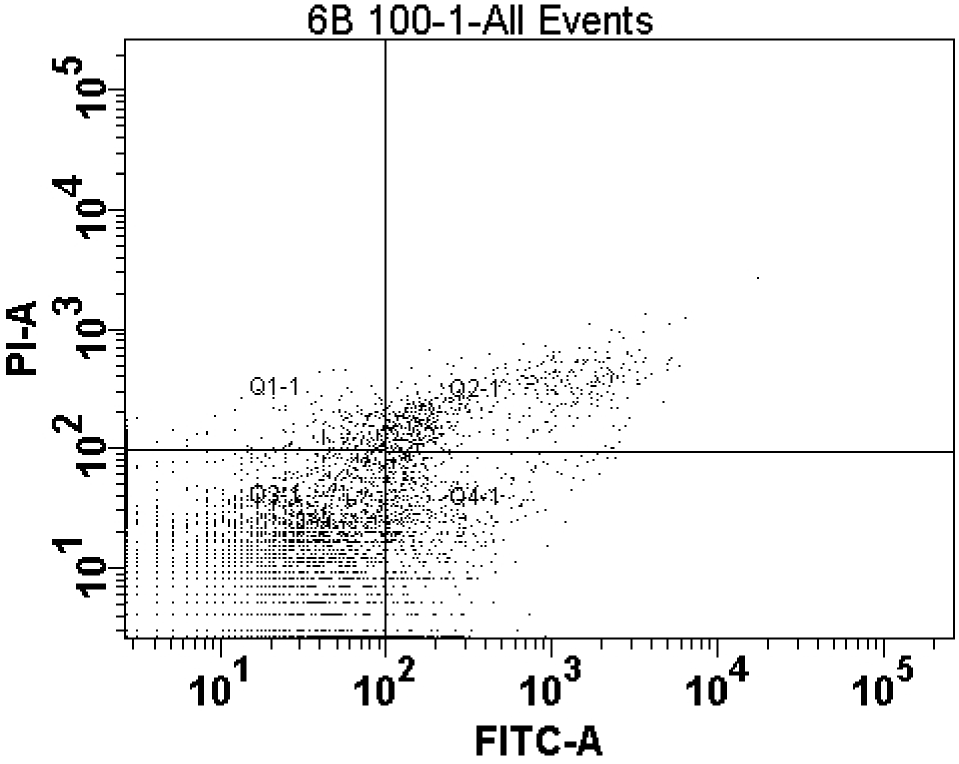
6B 100-1-All Events
PI-A
FITC-A
Q1-1
Q2-1
Q3-1
Q4-1
10^1
10^2
10^3
10^4
10^5

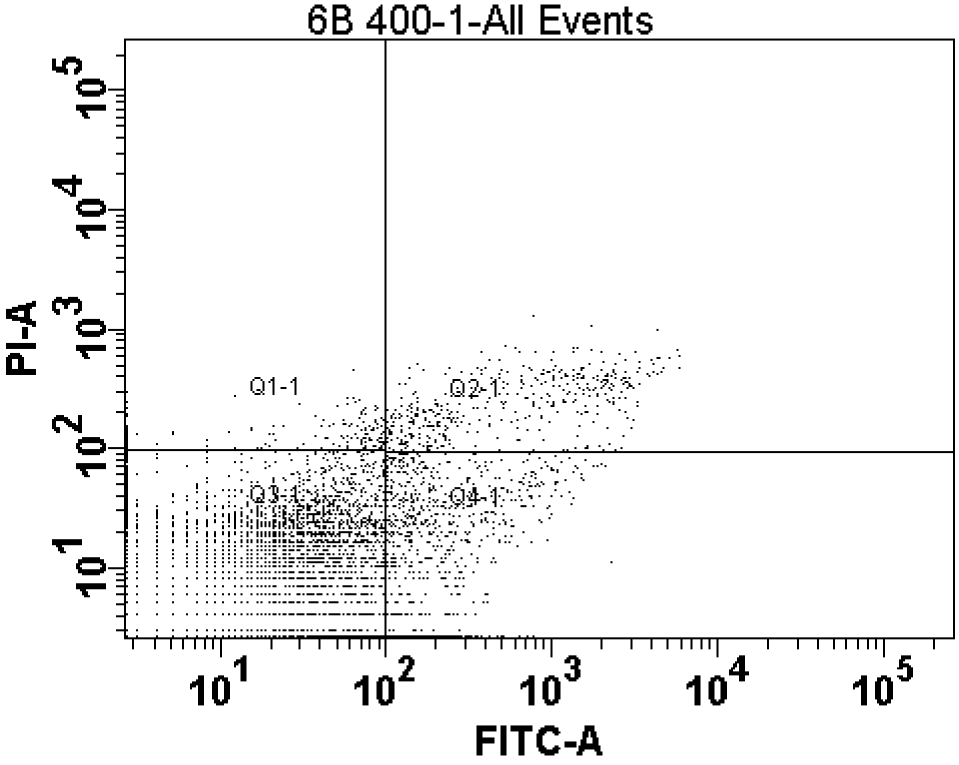
6B 400-1-All Events
PI-A
FITC-A
Q1-1
Q2-1
Q3-1
Q4-1
10^1
10^2
10^3
10^4
10^5

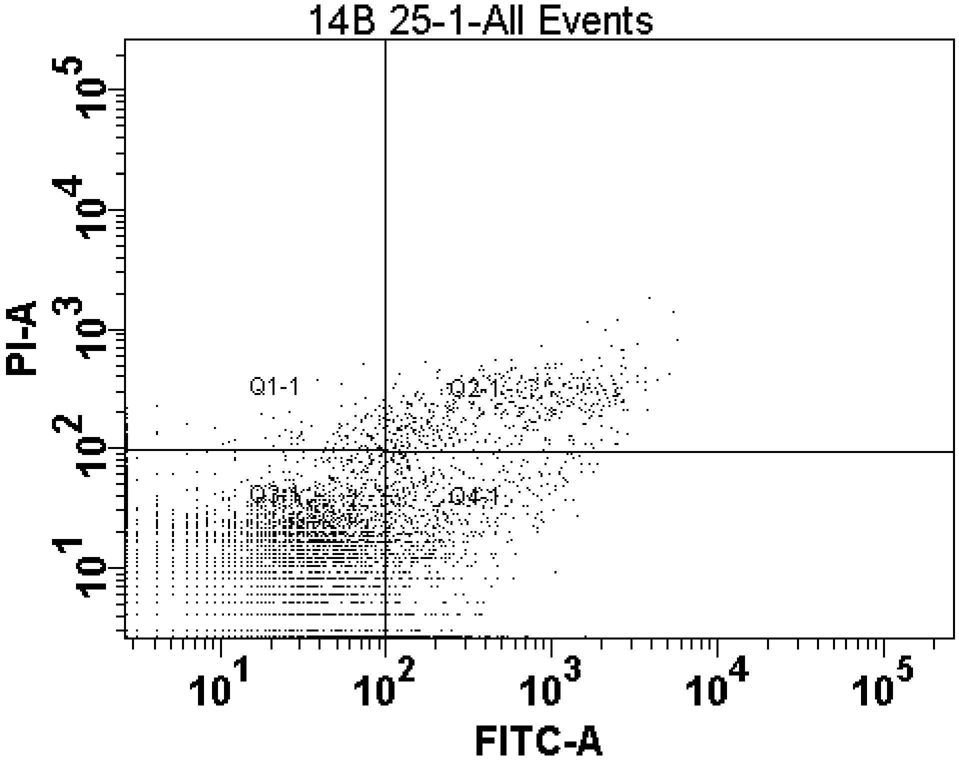
14B 25-1-All Events
PI-A
FITC-A
Q1-1
Q2-1
Q3-1
Q4-1

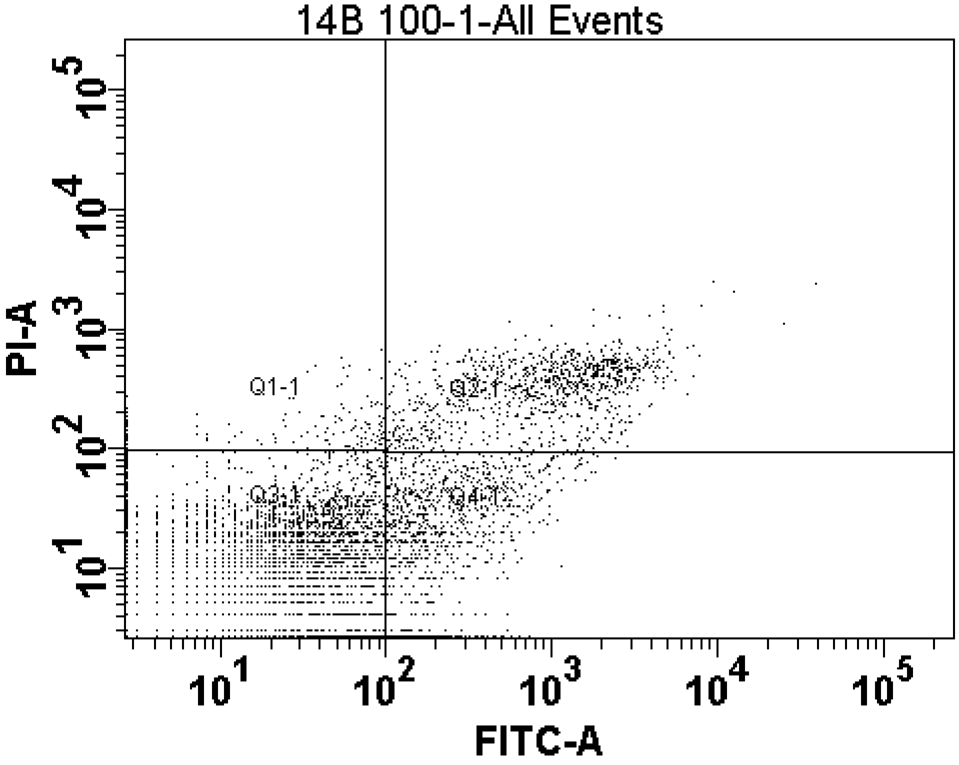
14B 100-1-All Events
PI-A
FITC-A
Q1-1
Q2-1
Q3-1
Q4-1

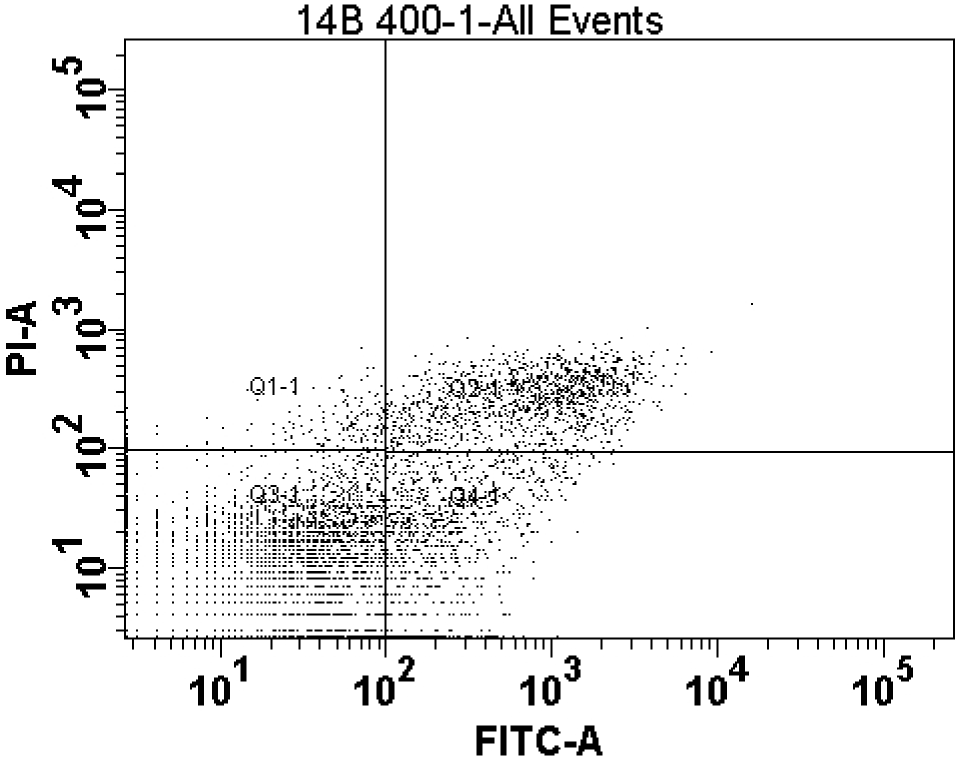
14B 400-1-All Events
PI-A
FITC-A
Q1-1
Q2-1
Q3-1
Q4-1

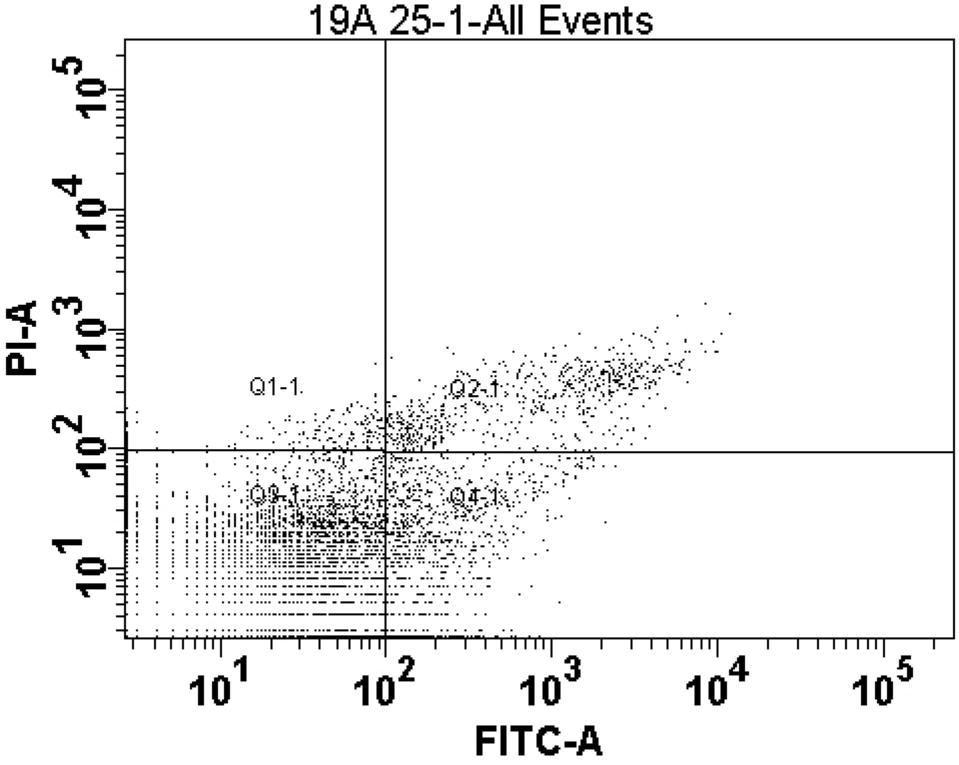
19A 25-1-All Events
PI-A
FITC-A
Q1-1
Q2-1
Q4-1

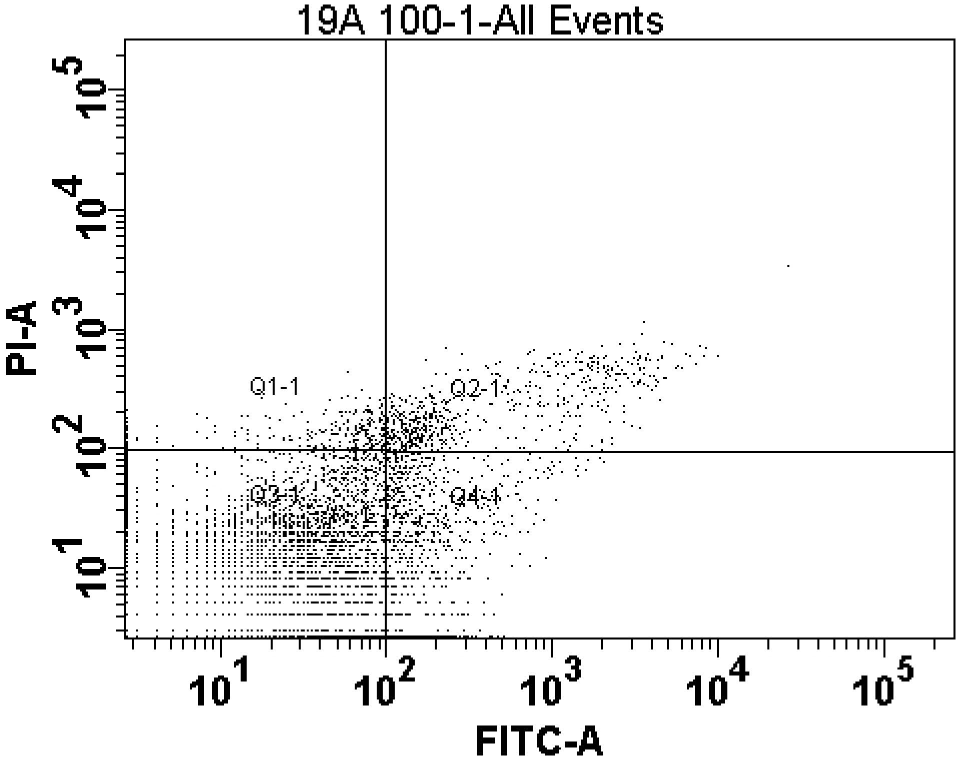
19A 100-1-All Events
PI-A
FITC-A
Q1-1
Q2-1
Q3-1
Q4-1
10^1
10^2
10^3
10^4
10^5

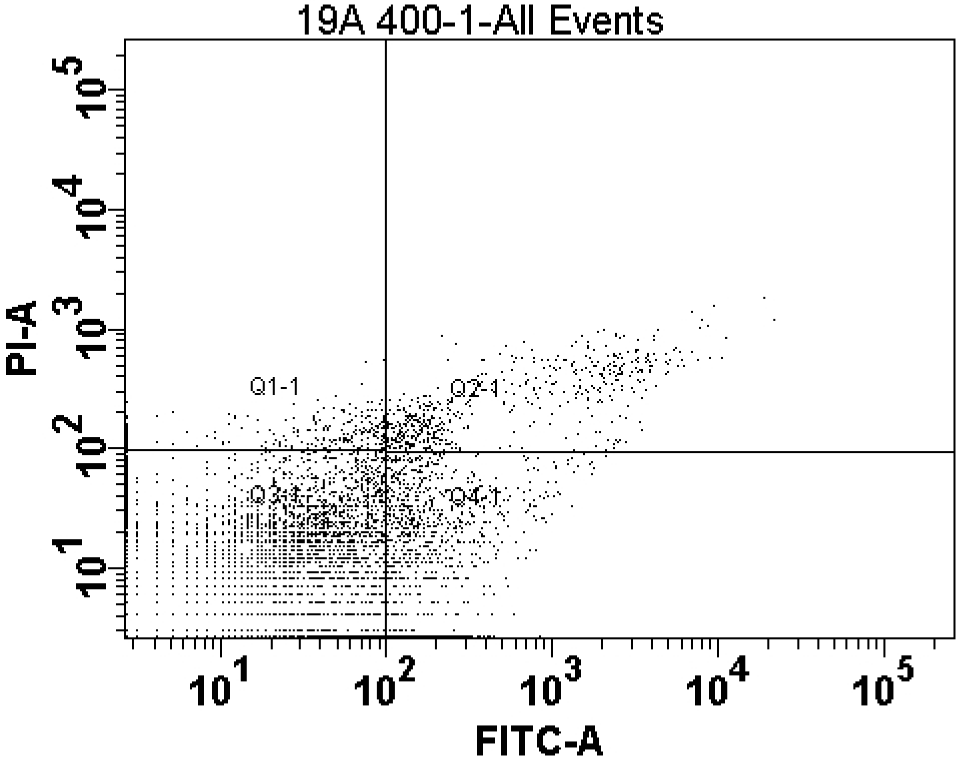
19A 400-1-All Events
PI-A
FITC-A
Q1-1
Q2-1
Q3-1
Q4-1
10^1
10^2
10^3
10^4
10^5
